# Therapeutic Options in Alzheimer’s Disease: From Classic Acetylcholinesterase Inhibitors to Multi-Target Drugs with Pleiotropic Activity

**DOI:** 10.3390/life14121555

**Published:** 2024-11-26

**Authors:** Ramón Cacabelos, Olaia Martínez-Iglesias, Natalia Cacabelos, Iván Carrera, Lola Corzo, Vinogran Naidoo

**Affiliations:** EuroEspes Biomedical Research Center, International Center of Neuroscience and Genomic Medicine, Bergondo, 15165 Corunna, Spain; epigenetica@euroespes.com (O.M.-I.); serviciodocumentacion@euroespes.com (N.C.); biotecnologiasalud@euroespes.com (I.C.); analisis@euroespes.com (L.C.); neurociencias@euroespes.com (V.N.)

**Keywords:** Alzheimer’s disease, drug development, epigenetic drugs, multi-target drugs, natural bioproducts, nosustrophine, pathogenic pathways, pharmacogenetics

## Abstract

Alzheimer’s disease (AD) is a complex/multifactorial brain disorder involving hundreds of defective genes, epigenetic aberrations, cerebrovascular alterations, and environmental risk factors. The onset of the neurodegenerative process is triggered decades before the first symptoms appear, probably due to a combination of genomic and epigenetic phenomena. Therefore, the primary objective of any effective treatment is to intercept the disease process in its presymptomatic phases. Since the approval of acetylcholinesterase inhibitors (Tacrine, Donepezil, Rivastigmine, Galantamine) and Memantine, between 1993 and 2003, no new drug was approved by the FDA until the advent of immunotherapy with Aducanumab in 2021 and Lecanemab in 2023. Over the past decade, more than 10,000 new compounds with potential action on some pathogenic components of AD have been tested. The limitations of these anti-AD treatments have stimulated the search for multi-target (MT) drugs. In recent years, more than 1000 drugs with potential MT function have been studied in AD models. MT drugs aim to address the complex and multifactorial nature of the disease. This approach has the potential to offer more comprehensive benefits than single-target therapies, which may be limited in their effectiveness due to the intricate pathology of AD. A strategy still unexplored is the combination of epigenetic drugs with MT agents. Another option could be biotechnological products with pleiotropic action, among which nosustrophine-like compounds could represent an attractive, although not definitive, example.

## 1. Introduction

In the last 100 years, 217,790 papers have been published on Alzheimer’s disease (AD), yet only eight drugs have been approved for its treatment. Seven were approved in the West by the US Food and Drug Administration (FDA) (Tacrine, introduced in 1945 as an efficient anticholinesterase agent and approved for AD treatment in 1993; Donepezil in 1996; Rivastigmine in 2000; Galantamine in 2001; Memantine in 2003; Aducanumab in 2021; Lecanemab in 2023) and one (Huperzine A) approved in China in 1994. These drugs belong to three different pharmacological categories: acetylcholinesterase inhibitors (AChEIs) (Tacrine, Donepezil, Rivastigmine, Galantamine), N-methyl-D-aspartate (NMDA) receptor antagonist (with glutamatergic-related anti-excitotoxic activity) (Memantine), and anti-amyloid monoclonal antibodies (Aducanumab, Lecanemab). Concerns about its hepatotoxicity led Tacrine to be withdrawn from the market in 2013; and the production of Aducanumab has recently been discontinued by the manufacturer. A new monoclonal antibody (Donanemab) was approved by the FDA for the treatment of adults with early symptomatic AD on 3 July 2024. Twenty years have passed since the introduction of memantine and the approval of Aducanumab without any new product being approved for treating AD [1,2,3].

Approximately 60% of AD patients take over 10 medications daily. Pharmaceutical spending on AD accounts for 15–30% of the direct costs associated with the disease. In 2020, the Alzheimer’s Association estimated that the total healthcare costs for Alzheimer’s and other dementias in the U.S. were $305 billion, with $51 billion attributed to direct pharmaceutical costs [4]. The total cost of dementia in Europe was estimated to be €250 billion in 2018, with approximately €10–15 billion attributed to pharmaceutical expenses [5]. Pharmaceutical spending on AD in Japan was estimated at $1.5 billion annually [6]. According to a market research report, the global AD drug market was valued at approximately $6.7 billion in 2020, with significant contributions from the USA, EU, and Japan [7].

**Figure 1:**
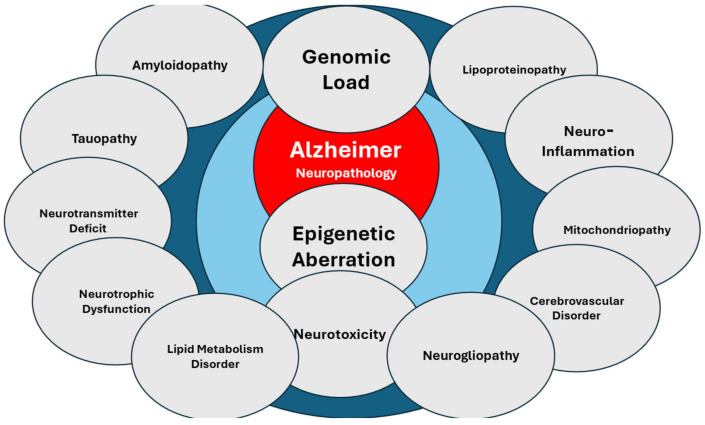
Confluent pathogenetic cascades and risk factors contributing to the phenotypic definition of Alzheimer’s disease.

This situation requires a rethinking of objectives in AD treatment. Current medications focus on two pathogenic components of the disease: (i) AChEIs aim to increase cholinergic neurotransmission based on the assumption of a cholinergic deficit responsible for the memory impairment present in AD [3], and (ii) monoclonal antibodies represent a modality of immunotherapy to clear the beta-amyloid protein (Aβ) deposits present in the senile plaques that flood the brains of AD patients [8]. However, in both cases, the approach to the disease is partial. The pathogenesis of AD is much more complex, suggesting that a more comprehensive approach may be necessary for effective treatment (Figure 1).

**Figure 2:**
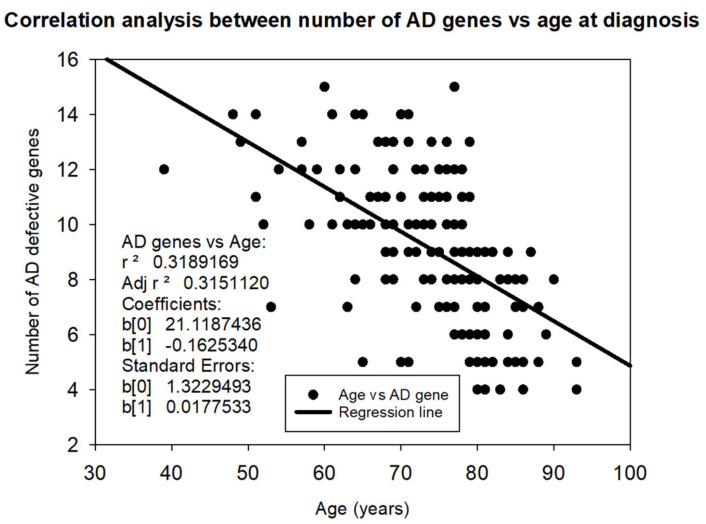
Correlation analysis between number of mutant AD genes per patient and age at diagnosis.

The various risk factors contributing to the convergent pathogenic cascades in AD include the following: (i) genetic factors: over 100 defective genes associated with AD have been identified in the human genome; 60% of AD patients carry more than 10 pathogenic variants; and the genetic load anticipates the age of risk, accelerates the course of the disease, and conditions the response to pharmacological treatment [9,10,11] (Figure 2); (ii) epigenetic factors: a multitude of changes in the epigenome (such as DNA methylation, chromatin and histone aberrations, and non-coding RNA dysfunction) alter gene expression, potentially leading to premature brain neurodegeneration [12,13,14,15,16]; (iii) cerebrovascular component: more than 80% of dementias in people over 70 years of age present clear cerebrovascular involvement, giving the dementia the character of a mixed clinical entity [17,18,19,20,21]; (iv) amyloidopathy: AD is a cerebral amyloidopathy with phenotypic expression of extracellular Aβ deposits in senile plaques that accumulate throughout the disease [22,23,24]; (v) tauopathy: AD shows a tauopathic component represented by intraneuronal neurofibrillary tangles (NFTs), resulting from the hyperphosphorylation of Tau protein [25,26,27]; (vi) neurogliopathy-related neuroinflammation [28]; (vii) glymphatic dysfunction: AD-related neuropathology may coexist with cerebral small vessel disease and glymphatic dysfunction, which contribute to neurodegeneration and cognitive impairment [29]; (viii) mitochondriopathy: alterations in mitochondrial function alter the energy metabolism of neurons [30]; (ix) oxidative stress: the genesis of reactive oxygen species (ROS), with free radical formation and lipid peroxidation, induces oxidative reactions that damage neurons [31,32,33]; (x) lipid alterations and disruption of lipid rafts: changes in the structure and composition of neuronal membranes become necropathic points that alter the function of membrane receptors and interneuronal communication [34,35,36]; (xi) neurotrophic dysfunction: failure in the neuronal damage repair systems due to a deficit of neurotrophic factors [37,38]; and (xii) neurotransmission deficits: neuronal loss, axonal damage, and dendritic dearborization reduce interneuronal communication due to the failure of various neurotransmission systems (acetylcholine, norepinephrine, dopamine, serotonin, histamine, glutamate, GABA, neuropeptides) [39,40,41,42,43].

Given the failure of the current limited forms of therapeutic intervention, an old school of thought suggests shifting from monotherapy to polytherapy and/or developing new treatments with multi-target drugs that act on different targets involved in the pathogenesis of AD. While this approach is only a partial solution, it may represent a slight advance in the search for better therapeutic solutions until the primary causes of the disease are definitively identified.

Over the past decade, more than 10,000 new compounds with potential action on some pathogenic components of AD have been tested, as well as over 1000 drugs with potential multi-target function (Tables S1, S2, and S4); however, unfortunately, none of these products have demonstrated sufficient efficacy to merit entering the clinical phase [1,44].

Multi-target (MT) drugs for AD aim to address the complex and multifactorial nature of the disease by acting on multiple pathological pathways. This approach has the potential to offer more comprehensive benefits than single-target therapies, which may be limited in their effectiveness due to the intricate pathology of AD.

The main objective of this article has been to review the progress made in the search for new treatments for AD in the last 10 years, with special interest in drugs catalogued as multi-target in PubMed and included in PubChem. An incursion is also made into the world of epigenetic drugs, as a future strategy, with special mention to those epidrugs with a potential effect on cognitive functions, as well as a brief description of their pharmacogenetic complexity; and, finally, the pleiotropic properties of a new biotechnological product (nosustrophin) are described, as a paradigmatic example of a prophylactic/preventive strategy in AD.

## 2. Drug Development

The total estimated annual investment in AD pharmaceutical development in the USA is approximately $5.95 billion, including substantial contributions from federal funding (NIH: $3.1 billion) [45], industry investment ($2.8 billion) [46], and non-profit organizations ($50 million) [4]. This represents approximately 12% of pharmaceutical expenditure in clinical care and reflects the significant resources dedicated to addressing this critical public health challenge. The total estimated annual investment in pharmaceutical development for AD in four representative European countries is as follows: Germany, €260 million; France, €190 million; Sweden, €95 million; and the United Kingdom, €268 million. Since 2000, database queries in PubMed, Web of Science, and Scopus indicate that out of 190,000 papers published on AD, more than 60,000 are from US research centers, 15,000 from China, 10,000 from the UK, 9000 from Germany, 8000 from France, and 3000 from India. Of all papers published worldwide on AD, approximately 20–25% report on results related to preclinical drug research in AD models or clinical trials of drugs either in development or already approved by the FDA [1,8,44,47].

Drugs and bioproducts studied during the last two decades in in vitro and in vivo AD models can be conventionally classified into at least 10 categories. Furthermore, 25–30% of all products studied are compounds derived from natural sources with pleiotropic activity; 20–25% are anti-amyloid agents with some capacity to clear Aβ deposits that accumulate extracellularly in neuritic/senile plaques; 15–20% of the reported products act as neurotransmitter enhancers; 12–15% are novel drugs designed to act on new targets; 10–15% are commonly used drugs that have been sought for some possible positive effects in AD; 6–8% are anti-tau drugs aimed at preventing hyperphosphorylation of the tau protein and the formation of intracellular neurofibrillary tangles; 3–5% are multi-target drugs with potential effects on several pathogenic pathways of AD; and the remaining 5–8% of drugs include a diverse mix of products, such as anti-inflammatory drugs (1–2%), new agents for stem cell therapy (1.85%), peptides with potential neuroprotective activity (1.5%), nanotechnology products with a specific therapeutic effect or adjuvants to facilitate the intracerebral penetration of other drugs (1.45%), and some other products (1%) of lesser relevance [44]. Despite their dubious efficacy, during the last decade, the vast majority of new chemical agents reported were neurotransmitter enhancers, 70–80% of which were cholinergic drugs, 15% glutamatergic agents, 8% serotonergic system regulators, 5% dopaminergic neurotransmission enhancing products, 3% histaminergic receptor regulating agents, 1% GABAergic modulators, and 1% adrenergic mechanism regulators [1,44].

Among the cholinergic drugs, AChEIs predominate, accounting for 75–80%, some of which are chemical structures derived from donepezil, tacrine, rivastigmine, and galantamine; 3–5% are butyrylcholinesterase inhibitors (BuChEIs); 3% are dual inhibitors of AChE and BuChE; 2–3% are dual inhibitors of AChE and monoamine oxidase (MAO); 6% are muscarinic receptor agonists; and 8% are nicotinic receptor agonists. Among the catecholaminergic agents, dopamine D1, D2/3, and D5 receptor agonists, and MAO-A (MAOAIs) and MAO-B inhibitors (MAOBIs) predominate. Moreover, 5-HT2A, 5-HT4, and 5-HT7 receptor agonists and 5-HT3 and 5-HT6 receptor antagonists are the most frequently developed serotonergic agents for AD. Among the glutamatergic agents, the most predominant are NMDA receptor antagonists (memantine-related chemicals, NMDA inhibitors, modulators of AMPA receptors and metabotropic glutamate receptors, glycine transporter 1 (GlyT1) inhibitors, glutamate modulators, and vesicular glutamate transporter inhibitors). The most studied GABAergic drugs are the allosteric modulators of GABA-A receptors and the GABA-A and GABA-B receptor agonists. Several histamine H3 receptor antagonists and other newer-generation antihistamines have been investigated. Among adrenergic drugs, agonists of β1 adrenergic receptors (ADRB1) and α2 adrenergic receptors (α2ARA) and selective antagonists of α2C adrenoceptors were the most commonly studied, with little impact in AD models [1,44].

## 3. Multi-Target Drugs

Although not yet well classified, in the international scientific literature, over 1000 drugs could be classified as multi-target (MT) drugs, of which fewer than 200 have documentation supporting their value as a potential AD treatment [1,44,48,49] (Table S4). A list of 62 MT compounds has been proposed after the computerized analysis of 7905 reports published from 1981 to 2022 [50].

The sine qua non condition of any MT drug is that it is capable of acting simultaneously on two or more pathogenic pathways associated with AD [49]. Most MT drugs, thus far, show a dual effect as AChEIs and BuChEIs with anti-amyloidogenic activity, biometal chelating effects, antioxidant activity, neuroprotection, and modest cognitive improvements in different AD models. Some of these novel drugs are complex derivatives of classical AChEIs; others are strictly novel drugs acting on new molecular mechanisms involved in the pathogenesis of AD; and the vast majority of the most pleiotropic products belong to the category of bioproducts derived from natural sources [1,44,51,52,53,54]. Four biologically active scaffolds, including Curcumin, Resveratrol, Chromone, and Indole, in hybrid formulations, show MT effects influencing AChE, BuChE, MAO-A, MAO-B, 5-HT4, SERT, β-amyloid self-aggregation, and radical scavenging activity [55]. Another set of novel MT drugs concentrates on β-secretase (BACE1), glycogen synthase kinase 3β (GSK-3β), and AChEIs as attractive therapeutic targets [56].

### 3.1. Tacrine Derivatives

Over 1000 tacrine derivatives have been developed since 1993, when tacrine was approved for the treatment of AD. Structural modifications in the tacrine molecule served to develop novel candidates with MT activity against some AD hallmarks (β-amyloid deposition, tau protein hyperphosphorylation, N-methyl-D-aspartate-receptor-related excitotoxicity, AChE, BuChE, MAO-A, MAO-B, secretases) (Table S1). Tacrine-based derivatives with heterocyclic structures (tetrahydroquinolone, dihydroxypyridine, coumarin, chromene, triazole, pyrazole, arylisoxazole, dipicolylamine) and many different tacrine hybrids have been developed (Tables S1 and S4), some of them devoid of toxic effects and with superior anti-AD activity compared to tacrine [57,58,59,60].

#### 3.1.1. Tacrine-Based Cyclopentapyranopyridine– and Tetrahydropyranoquinoline–Kojic Acid Derivatives

A novel series of tacrine-based cyclopentapyranopyridine– and tetrahydropyranoquinoline–kojic acid derivatives show anti-cholinesterase activity (AChE>BChE). Tetrahydropyranoquinoline–kojic acid derivatives show weaker AChE inhibitory activity than Cyclopentapyranopyridine–kojic acid derivatives. The highest anti-AChE activity is observed with the compound 10-amino-2-(hydroxymethyl)-11-(4-isopropylphenyl)-7,8,9,11-tetrahydro-4H-cyclopenta[b]pyrano[2′,3′: 5,6]pyrano [3,2-e]pyridin-4-one, which bears a 4-isopropylphenyl moiety and a cyclopentane ring. Some compounds of this class show moderate neuroprotective properties, especially against H_2_O_2_-induced cytotoxicity [61].

#### 3.1.2. Tetrahydroaminoacridine (THA)–Ferulic Acid Hybrids

These compounds combine AChE inhibitory activity with antioxidant and anti-amyloid properties. They target cholinesterase, Aβ aggregation, and oxidative stress, thereby inhibiting Aβ-self-aggregation and improving cognition in scopolamine-induced models [62].

#### 3.1.3. Tacrine–Resveratrol Fused Hybrids

Tacrine–resveratrol fused hybrids inhibit human AChE, are modulators of Aβ self-aggregation, and display immuno-modulatory and anti-inflammatory activity in neuronal and glial AD cell models [63].

#### 3.1.4. Tacrine–Benzofuran Hybrids

Novel tacrine–benzofuran hybrids are potent AChE inhibitors, with metal (Fe^2+^, Cu^2+^) chelating ability, anti-oxidant activity, and neuroprotective effects against Aβ1-42-induced toxicity [64].

#### 3.1.5. Tacrine–Hydroxyphenylbenzimidazole Hybrids

Tacrine–hydroxyphenylbenzimidazole (TAC-BIM) hybrid molecules show AChE inhibitory activity, radical scavenging activity, metal chelating ability, strong inhibition of self-induced and Cu^2+^-induced Aβ aggregation, and also inhibition of Aβ- and Fe^2+^/AscH^−^-induced neurotoxicity [65].

#### 3.1.6. Tacrine–Deferiprone Hybrids

Hybridization of tacrine with the metal chelating drug deferiprone yields hybrid compounds with AChE inhibitory capacity; inhibition of self-/Cu^2+^-induced Aβ aggregation; metal chelating capacity for Fe^2+^, Cu^2+^, and Zn^2+^; and free radical scavenging ability. The compounds with 2-hydroxypropyl linkers show neuroprotective effects in neuroblastoma cells treated with Aβ1-42 [66].

#### 3.1.7. Multifunctional Tacrine–Donepezil Hybrids

A series of tacrine–donepezil hybrids, resulting from the fusion of tacrine and the benzylpiperidine moiety of donepezil with a hydrazone group, show dual inhibitory activity toward AChE and BuChE, neuroprotection against H_2_O_2_-induced cell death in a human neuroblastoma cell line, and blood–brain barrier (BBB) permeability on HBEC-5i cells [67].

#### 3.1.8. Conjugates of Tacrine with 1,2,4-Thiadiazole Derivatives

Conjugates of tacrine with 1,2,4-thiadiazole derivatives, with pentylaminopropene and pentylaminopropane as linkage spacers, are potent BuChE inhibitors and effective blockers of AChE-induced Aβ-aggregation, with high radical-scavenging capacity and lower potency against carboxylesterase [68].

#### 3.1.9. Conjugates of Tacrine and Salicylamide: Salicylimine Derivatives

Tacrine–Salicylimine conjugates are selective BuChE inhibitors and also inhibitors of AChE. **Aalicylimine derivatives** are the most active conjugates, with weak inhibition of carboxylesterase. These hybrids inhibit AChE-induced Aβ-aggregation and Aβ42 self-aggregation. In addition, they show BBB permeability, free radical scavenging activity and are effective chelators of Cu^2+^, Fe^2+^, and Zn^2+^ [69].

#### 3.1.10. Tacrine Conjugates with 2-Arylhydrazinylidene-1,3-Diketones

The combination of tacrine and antioxidant 2-tolylhydrazinylidene-1,3-diketones with an aminoalkylene linker creates three groups of hybrid compounds with potential MT activity in AD models. These compounds inhibit AChE and BuChE and show weak inhibition of carboxylesterase. The hybrids block AChE-induced Aβ aggregation and show metal chelating ability for Cu^2+^, Fe^2+^, and Zn^2+^ and high antiradical activity [70].

#### 3.1.11. Methylene-Linked 1,2,3,4-Tetrahydrobenzo[h][1,6]naphthyridine-6-chlorotacrine Hybrids

Methylene-linked 1,2,3,4-tetrahydrobenzo[h][1,6]naphthyridine-6-chlorotacrine hybrids are potent inhibitors of AChE and BuChE and behave as Aβ42 and tau antiaggregating agents, with good brain permeability. The most potent AChEI of this series, stronger than the reference compound 6-chlorotacrine, shows potent self-induced Aβ1–42 antiaggregating and tau antiaggregating activities [71].

#### 3.1.12. Quinolinetrione–Tacrine Hybrids

Changes in the molecule of tacrine–quinone hybrids by replacing the naphthoquinone scaffold with 2,5,8-quinolinetrione yields quinolinetrione–tacrine hybrids, with potent AChE and BuChE inhibitory activity, antioxidant effects, and anti-amyloid aggregation properties [72].

#### 3.1.13. Tacrine–Selegiline Hybrids

Novel tacrine–selegiline hybrids are potent AChE, BuChE, MAO-A, and MAO-B inhibitors, penetrate the BBB, and improve cognitive function in mice treated with scopolamine [73].

#### 3.1.14. Tacrine–Flavone Hybrids

Tacrine–Flavone hybrids, represented by the most active compound AF1, exhibit inhibitory effects on AChE and MAO-B enzymes. Compound AF1 does not show toxicity in rats, with an LD_50_ value of 64 mg/kg bodyweight [74].

#### 3.1.15. Capsaicin–Tacrine Hybrids

Capsaicin–Tacrine hybrids inhibit AChE, BuChE, and β-secretase-1 (BACE-1) enzymes [75].

### 3.2. Donepezil Derivatives

Over the past 25 years, hundreds of molecular structures related to donepezil have been designed, synthesized, and validated, some of which showed MT activity [76] (Tables S2 and S4).

#### 3.2.1. E2020-NOH

E2020-NOH is a derivative of donepezil with additional nitric-oxide-releasing properties. It targets cholinesterases, provides neuroprotection, and modulates cerebral blood flow [77].

#### 3.2.2. 1-Aryldonepezil Analogs

1-Aryldonepezil analogs are synthesized through a palladium/PCy3-catalyzed Suzuki reaction. The 4-tert-butylphenyl analog exhibits good inhibitory potency against AChE and BuChE, with neuroprotective effects on H_2_O_2_-induced cell injury [78].

#### 3.2.3. Multifunctional Aromatic Amine Hybrids of Donepezil

Modifications in the active moiety of donepezil (DNP), benzylpiperidine, linked to the neurotransmitter phenylethylamine through a squaramide bond and substitutions in the benzene rings produce a series of MT hybrid compounds (DNP-phenylethylamine, DNP-benzylamine, and DNP-aniline hybrids) with AChE inhibitory activity and neuroprotective effects in the SH-SY5Y cell line [79].

#### 3.2.4. Racemic Trans Propargylamino-Donepezil

Propargylaminodonepezil (PADPZ) results from the fusions of two indane derivatives, donepezil and rasagiline, with structural analogy. The synthesis of racemic trans-PADPZ shows inhibitory activities towards human AChE and MAO-B enzymes, recapitulating the original effects of donepezil and rasagiline, respectively [80].

#### 3.2.5. Cinnamoyl-N-Acylhydrazone-Donepezil Hybrids

MT Cinnamoyl-N-acylhydrazone-donepezil hybrids result from the molecular hybridization of 1-benzyl-4-piperidine from donepezil and the cinnamoyl subunit from curcumin, using a N-acylhydrazone fragment as a spacer subunit. These hybrid compounds exhibit moderate inhibitory activity towards AChE, free radical scavenging activity, and protection of neuronal cells against damage induced by tert-Butyl hydroperoxide (t-BuOOH) and 6-Hydroxydopamine (6-OHDA). ROS formation is prevented by some of these compounds through indirect antioxidant activity, with increased intracellular levels of GSH and inhibition of Aβ1-42- and 3-NP-induced neurotoxicity [81].

#### 3.2.6. Phenothiazine/Donepezil-like Hybrids

Linking a phenothiazine moiety (antioxidant) with N-benzylpiperidine or N-benzylpiperazine fragments, which are the core substructures of donepezil, generates a new series of MT hybrid molecules with AChE and BuChE inhibitory activity, antioxidant properties, anti-Aβ1-40 aggregation capacity, and inhibition of fatty acid amide hydrolase activity [82].

#### 3.2.7. N-Benzyl-piperidinyl-aryl-acylhydrazone Derivatives

MT N-benzyl-piperidine-aryl-acylhydrazone derivatives are molecules that combine the N-benzyl-piperidine subunit of donepezil, the substituted hydroxy-piperidine fragment of the AChE inhibitor LASSBio-767, and an acylhydrazone linker into a single molecule. These hybrids function as AChEIs and exhibit anti-inflammatory activity against Aβ oligomer (AβO)-induced neuroinflammation and neuroprotective effects against AβO-induced neurodegeneration [83].

#### 3.2.8. Donepezil-Arylsulfonamide Hybrids

The combination of an aryl-sulfonamide with a benzylpiperidine moiety, the pharmacophore of donepezil, or its benzylpiperazine analog yields a new series of donepezil-arylsulfonamide hybrids with MT activity: AChE inhibitory activity, prevention of Aβ aggregation, Aβ-induced cell toxicity, and good BBB permeability [84].

#### 3.2.9. Vilazodone–Donepezil Chimeras

Merging the 5-HT1A receptor partial agonist and serotonin transporter inhibitor vilazodone and donepezil generates a series of vilazodone–donepezil chimeric derivatives with triple-target activity against AChE, 5-HT1A receptors, and the serotonin transporter. These chimeras might be potentially useful as a prospective therapeutic intervention for mood disorders in dementia [85].

### 3.3. Derivatives of Other AChE Inhibitors

#### 3.3.1. Rivastigmine–Hydroxyphenylbenzimidazole Hybrids

The conjugation of the active moiety of rivastigmine with 2 isomeric hydroxyphenylbenzimidazole results in novel hybrids of rivastigmine with anti-AD properties, including AChE and BuChE inhibitory activity, metal chelation, antioxidation, and inhibition of Aβ aggregation [86].

#### 3.3.2. Apigenin–Rivastigmine Hybrids

Novel apigenin–rivastigmine hybrids are potent antioxidants and reversible inhibitors of AChE and BuChE, with selective metal-chelating properties. They effectively inhibit both self-mediated and Cu^2+^-mediated Aβ1-42 aggregation. Some of these compounds also show remarkable neuroprotective effects and hepatoprotective activity, favorable BBB penetration, and protection against dyskinesia and Aβ1-40-induced vascular injury in zebrafish models. They also exhibit a capacity for improvement in scopolamine-induced memory impairment [87].

#### 3.3.3. Galantamine–Memantine Hybrids and Galantamine–Curcumin Hybrids

Galantamine–Memantine hybrids and Galantamine–Curcumin hybrids have been developed, but their effects are not superior to those of the individual drugs [88].

#### 3.3.4. Huperzine A

Huperzine A acts as an AChE inhibitor and has neuroprotective properties. It targets AChE, oxidative stress, and glutamate excitotoxicity [89].

### 3.4. Memantine Derivatives

Memantine (3,5-dimethyladamantan-1-amine) is a noncompetitive N-methyl-D-aspartate receptor (NMDAR) antagonist for treating moderate-to-severe AD. A few memantine hybrids have been designed and synthesized as potential MT agents for AD.

#### 3.4.1. Tacrine–Adamantanes Hybrids

Hybrids of 7-methoxytacrine (7-MEOTA) and amantadine, linked by methylene-thiourea or methylene-urea tethers, show AChE and BuChE inhibitory activity, modulation of NMDA receptors, and inhibition of Aβ1-40 aggregation [90]. Benzo-fused analogs of the adamantylamine scaffold are potent NMDAR antagonists; and fluorobenzohomoadamantanamine displays more potent effects than memantine and amantadine [91].

#### 3.4.2. Galantamine–Memantine Conjugates

Galantamine–memantine conjugates exhibit NMDAR antagonism and AChE inhibition [90]. ARN14140, a Memantine/Galantamine-based MT compound, demonstrates neuroprotective activity against Aβ25–35-induced neurotoxicity. This compound reduces Aβ25–35-induced memory deficits, prevents increased lipid peroxidation, TNFα levels, inflammatory events, and a decrease in hippocampal synaptophysin levels, thus protecting synaptic function. ARN14140 also reduces Aβ25–35-induced neurotoxicity by reducing hippocampal Bax content and prevents Aβ25–35-induced cell loss in the mouse hippocampal CA1 region [92].

#### 3.4.3. Aminoadamantane–Carbazole/Tetrahydrocarbazole Hybrids

Hybrids of aminoadamantanes with carbazole and tetrahydrocarbazole are inhibitors of human AChE and equine serum BuChE, antagonists of NMDARs, and stimulators of microtubule assembly [93].

#### 3.4.4. Memantine–Antioxidant Hybrids

**Memantine–Ferulic Acid hybrids** show efficacy in reducing Aβ-induced neurotoxicity and oxidative stress, NMDAR-blocking activity, radical scavenger effects, and activation of the Nrf2-ARE pathway. This type of compound may stimulate the non-amyloidogenic pathway, reducing Aβ production [94].

**Memantine–Glutathione/Lipoic Acid hybrids** combine the NMDA receptor antagonism of memantine with the radical scavenging activity of glutathione (GSH) and (R)-α-lipoic acid (LA), preventing oxidative stress and cell damage. Some of these derivatives may also inhibit Aβ aggregation, protecting cells against Aβ-mediated cytotoxicity [95].

#### 3.4.5. Memantine–Polyamine Conjugates

Memantine–polyamine conjugates show potent NMDAR antagonism, superior to that of memantine, with strong reversible and voltage-dependent NMDA blocking activity and with inhibitory effects on GluN1/N2A and GluN1/N2B NMDARs [96].

#### 3.4.6. H_2_S-Releasing Memantine Prodrug

Carbon monoxide (CO), nitric oxide (NO), and hydrogen sulfide (H_2_S) are endogenous gasotransmitters, with important roles as antioxidants, neuromodulators, and anti-inflammatory agents. H₂S provides neuroprotective, anti-inflammatory, and antiapoptotic effects in addition to its NMDAR-modulating ability and regulation of intracellular calcium concentration [97].

#### 3.4.7. Dual P2X7-NMDA Receptor Antagonists

The P2X7 receptor (P2X7R) is an ATP-sensitive ion channel in glial cells, mediating K^+^ efflux and Ca^2+^/Na^+^ influx. cAMP response element-binding protein (CREB) activation inhibits the transcription of genes involved in microglia-related inflammatory responses and stimulates α-secretase, leading to the formation of soluble and non-toxic sAPPα. Microglial P2X7Rs are upregulated in AD, which triggers an inflammatory cascade that releases IL-1β, INF-γ, TNF-α, and other cytokines. P2X7Rs also contribute to release glutamate, which activates NMDARs. P2X7R inhibition reduces Aβ levels and protects neurons against Aβ toxicity. Amantadine or memantine moieties bound to a N’-arylcarbohydrazide core yield novel P2X7R-NMDAR antagonists that potentiate P2X7R antagonism, contributing to MT neuroprotection [90,98].

### 3.5. Diverse Chemical Derivatives with MT Effects

#### 3.5.1. Ladostigil

Ladostigil has cholinesterase and MAO inhibitory properties and neuroprotective effects. It targets cholinesterases (AChE, BuChE) and MAO enzymes and provides antioxidant and neuroprotective benefits [99,100]. Ladostigil prevents memory decline and suppresses the overexpression of genes encoding pro-inflammatory cytokines (TNFα, IL1β, IL6) in the brain and microglial cells. Ladostigil decreases *Egr1* gene expression and increases TNF-alpha-induced protein 3 (TNFaIP3), responsible for the suppression of cytokine release in microglial cells [101].

#### 3.5.2. M30

M30 acts as a multifunctional iron chelator, MAO inhibitor, and antioxidant. It targets iron dysregulation, MAO activity, and oxidative stress [102]. M30 is a first class of site-activated chelator with dual inhibition of AChE and MAO, designed to simultaneously target multiple pathogenic processes in AD without disrupting metal metabolism. The novel **Prochelator 2** is a selective and potent MAO-A inhibitor with moderate MAO-B inhibition and selective AChE inhibition. Prochelator 2 is activated by brain AChE, releasing M30, an effective chelator of metal ions (Fe^2+^, Cu^2+^, and Zn^2+^), suppressor of oxidative stress, inhibitor of MAO-A and MAO-B in the brain, and regulator of cerebral biometal dyshomeostasis. M30 is neuroprotective, with effects against Aβ generation, Aβ aggregation induced by metal ions, Aβ deposits in senile plaques, and intracellular NFT formation [103,104].

#### 3.5.3. Memoquin

Memoquin is a hybrid compound with cholinesterase inhibitory activity, inhibition of Aβ aggregation and secretase activity, and antioxidant properties [105,106,107,108].

#### 3.5.4. ASS234

ASS234 is a multi-target compound that inhibits cholinesterases and MAO enzymes and modulates Aβ aggregation [109].

#### 3.5.5. RS-0406

RS-0406 acts as a neuroprotective agent with Aβ aggregation inhibition and anti-inflammatory properties [110].

#### 3.5.6. Acridine

Acridine is the chemical structure found in several pharmaceutical categories, including antimalarials (quinacrine), antiseptics (acriflavine, proflavine), abortifacients (ethacridine), anti-cancer drugs (amsacrine, nitracrine), and anti-dementia drugs (tacrine). It has been proposed that acridine might be an MT drug against AD, acting on AChE, BuChE, dual specificity tyrosine kinase 1A (Dyrk 1A), Aβ, and prion protein (PrPC) [111].

#### 3.5.7. Indanone Derivatives

Indanone derivatives exhibit inhibitory activity against AChE, similar to donepezil, and inhibit Aβ aggregation (>80%), disaggregate Aβ fibrils formed through self-induced Aβ aggregation, and possess antioxidant activity [112].

#### 3.5.8. Chromone Derivatives

7-Hydroxy-chromone derivatives, with a pyridine moiety, are AChEIs and BuChEIs, showing neuroprotective activity against H_2_O_2_- and Aβ-induced neurotoxicity. These compounds also block self- and AChE-induced Aβ aggregation [113].

#### 3.5.9. Quinazolines

Quinazolines are bioactive heterocyclic compounds with inhibitory activity on β-amyloid, tau protein, cholinesterases, monoamine oxidases, and phosphodiesterases [114].

#### 3.5.10. Benzyl Pyridinium-2,4-dioxochroman Derivatives

Novel benzyl pyridinium-2,4-dioxochroman derivatives are potent AChEIs and anti-BuChEIs with neuroprotective effects, similar to quercetin, and inhibitory activity against Aβ peptide aggregation [115].

#### 3.5.11. 2,2′-Bipyridyl Derivatives

New 3,3′-diamino-2,2′-bipyridine derivatives are selective Cu^2+^ chelators that inhibit Cu^2+^-induced Aβ1-42 aggregation and self-induced Aβ1-42 aggregation. One of these compounds is neuroprotective against Aβ1-42 and Cu^2+^-treated Aβ1-42-induced cell damage and also reverses Aβ-induced memory impairment in animal models [116].

#### 3.5.12. 8-Hydroxyquinoline Derivatives

MT 8-hydroxyquinoline derivatives inhibit self-induced Aβ1-42 aggregation and oxidative-stress-related damage. They are biometal chelators, inhibiting Cu^2+^/Zn^2+^-induced Aβ1-42 aggregation, and show protective effects against H_2_O_2_ and good BBB penetration [117].

#### 3.5.13. Hybrid 8-Hydroxy Quinoline–Indole Derivatives

Hybrid 8-hydroxyquinoline–indole derivatives are inhibitors of self-induced and metal-ion-induced Aβ1-42 aggregation. The most potent hybrid compound shows over 82% inhibition of Cu^2+^-induced Aβ1-42 aggregation and nearly 90% inhibition of Zn^2+^-induced Aβ1-42 aggregation. Some compounds are effective in reducing protein aggregation in HEK-tau and SY5Y-APPSw cells [118].

#### 3.5.14. Lithium

In some models, lithium shows neuroprotective effects associated with the homeostatic modulation of mitochondrial dysfunction, oxidative stress, inflammation, and autophagy. Some of these effects are attributed to inhibition of GSK-3 and inositol 1,4,5-trisphosphate [119].

#### 3.5.15. Pregnenolone Derivatives

Pregnenolone derivatives inhibit AChE, BuChE, hCA-II, and self-mediated Aβ1-42 peptide aggregation [120].

#### 3.5.16. Phosphazine and Phosphazide Derivatives

Phosphazine and phosphazide derivatives are inhibitors of AChE and Aβ aggregation. The coumarin phosphazide derivative shows the best AChE inhibition selectivity index together with good inhibition ability against MMP-2 and self-induced Aβ1-42 aggregation. The most potent of these compounds specifically chelates metal and is permeable to BBB, with low toxicity on SH-SY5Y neuroblastoma cells. This product also improves cognitive performance, similarly to donepezil, in mice treated with scopolamine [121].

#### 3.5.17. N-Benzylpyrrolidine Derivatives

MT hybrids of N-benzyl pyrrolidine derivatives are inhibitors of AChE, BuChE, and BACE-1. Lead compounds show excellent brain permeation, strong PAS-AChE binding capability, neuroprotective effects against Aβ-induced stress, and potential disassembly of Aβ aggregates. They also exhibit antioxidant activity and improve cognitive dysfunction in the scopolamine-induced amnesia model, as demonstrated by the Y-maze test, and against Aβ-induced cognitive dysfunction, as shown by the Morris water maze test [122].

#### 3.5.18. Clioquinol-1-benzyl-1,2,3,6-tetrahydropyridine Hybrids

Clioquinol-1-benzyl-1,2,3,6-tetrahydropyridine hybrids show neuroprotection against okadaic acid-induced mitochondrial dysfunction and ROS damage, inhibition of AChE, metal chelating properties, modulation of AChE- and metal-induced Aβ aggregation, capacity to reduce p-Tau levels, improvement of cognitive and spatial memory in two AD models, and suppression of neuro-inflammation induced by Aβ1-42 in the cortex [123].

#### 3.5.19. 2-Substituted Benzo[d]oxazol-5-amine Derivatives

2-Substituted benzo[d]oxazol-5-amine derivatives are potent inhibitors of AChE, BuChE, and Aβ aggregation. They show good BBB permeability, neuroprotective properties, and improvement in cognitive function and spatial memory [124].

#### 3.5.20. MT Thiazolidinediones

Thiazolidinediones (TZDs) function as agonists of peroxisome proliferator-activated receptor gamma (PPARγ) and inhibitors of long-chain acyl-CoA synthetase family member 4 (ACSL4) [125]. PPARγ belongs to the peroxisome proliferator-activated receptor (PPAR) family, which also includes PPARα and PPARβ/δ. Endogenous polyunsaturated fatty acids (PUFAs) serve as natural low-affinity ligands for PPARγ, while TZDs are high-affinity exogenous activators. Upon binding of an agonist, PPARγ undergoes transactivation, leading to the positive regulation of cell metabolic homeostasis, with effects on cellular differentiation, insulin sensitization, adipogenesis, and fat storage. The anti-inflammatory effects of PPARγ are regulated by transrepression rather than transactivation. Activated microglia, reactive astrocytes, and proinflammatory cytokines are involved in neuroinflammation. Agonist-bound PPARγ inhibits neuroinflammation by antagonizing the function of inflammation-related transcription factors (nuclear factor-κB (NF-κB), activator protein-1 (AP-1), JAK/STAT, nuclear factors of activated T-cells (NFAT) through transrepression mechanisms [125,126]. Acyl-CoA synthetases (ACS) convert fatty acids to acyl-CoA-activated forms, which participate in energy metabolism and membrane structure. Non-activated free fatty acids are involved in the production of prostaglandins (PGs), leukotrienes (LTs), and thromboxane (TX). ACSLs integrate five ACSL isozymes (ACSL1, ACSL5, ACSL6, ACSL3, ACSL4). TZDs inhibit ACSL4 in a PPARγ-independent manner [127]. The first TZD, troglitazone, was approved in 1997 by the FDA for the treatment of type 2 diabetes and was withdrawn from the market in 2000 due to its hepatotoxicity. Rosiglitazone and pioglitazone were introduced into the market in 1999. Rosiglitazone was banned in Europe and the USA in 2010 because of the high risk of myocardial infarction. TZDs ameliorate neuroinflammation and ferroptosis in preclinical models of AD [126].

#### 3.5.21. 2-Propargylamino-naphthoquinone Derivatives

A series of 10 novel derivatives of 2-propargylamino-naphthoquinone are inhibitors of MAO-A, MAO-B, Aβ aggregation, and free-radical formation. They also show metal-chelating properties and anti-inflammatory effects [128].

#### 3.5.22. MT Tryptamine Derivatives

A series of tryptamine analogs show MT properties in AD models. Three compounds (SR42, SR25, SR10) display AChE inhibitory activity superior to donepezil. SR42 is also a MAO-B inhibitor with superior inhibitory effects compared to tryptamine; and SR22, SR24, and SR42 exhibit inhibition against the COX-2 enzyme. SR42 is a 4,5 nitro-benzoyl derivative of tryptamine [129].

#### 3.5.23. Deoxyvasicinone–Indole

Analogs of deoxyvasicinone–indole, resulting from the conjugation of the pharmacophores of deoxyvasicinone and indole, are inhibitors of AChE, BuChE, and Aβ aggregation. One of these compounds also shows inhibition of AChE-induced Aβ1-42 aggregation by 80% [130].

#### 3.5.24. Benzofurans

Benzofurans are oxygen-containing heterocycles found in a number of biological molecules. These chemical motifs display antibacterial, antifungal, antioxidant, antitumoral, anti-inflammatory, anticonvulsant, anti-HIV bioactivities, and anti-AD properties (neuroprotection, inhibition of Aβ fibril formation, BuChEI) [131]. Some novel benzofurans inhibit BuChE, show neuroprotective activity against Aβ1-42 oligomers, are selective CB2 ligands (CB2 inverse agonist), and have immunomodulatory effects, switching microglia from the pro-inflammatory M1 to the neuroprotective M2 phenotype [132].

#### 3.5.25. Ibudilast

Ibudilast is an MT phosphodiesterase inhibitor and toll-like receptor 4 (TLR4) antagonist that inhibits off-target kinases (IRAK1, GSG2). In some Asian countries, ibudilast is used to treat asthma and stroke. Ibudilast improves hippocampal-dependent spatial learning, memory deficits, and AD pathology in Fisher transgenic 344-AD rats. Ibudilast alters gene expression levels of the TLR and ubiquitin–proteasome pathways and inhibits IRAK1 activity by increasing expression of its negative regulator IRAK3, and by altering TRAF6 and other TLR-related ubiquitin ligase and conjugase levels [133].

#### 3.5.26. N-Cyclohexylimidazo[1,2-a]pyridine Derivatives

A series of newly synthesized substituted benzyl-1H-1,2,3-triazol-4-yl-N-cyclohexylimidazo[1,2-a]pyridin-3-amines demonstrate that most of the compounds exhibited moderate to potent inhibitory activity against BACE1 and BuChE and antioxidant properties. Compounds containing dichloro (2,3-Cl2 and 3,4-Cl2) groups on the benzyl pendant were identified as the most effective BACE1 inhibitors. These compounds also showed antioxidant activity and potential for metal chelation [134].

#### 3.5.27. 20(R)-Panaxadiol Derivatives

A series of novel 20(R)-panaxadiol derivatives with benzyl-substituted carbamate at the 3-OH position show neuroprotective effects, AChE inhibition, and a wide range of biological activities (inhibition of apoptosis, induction of tau hyperphosphorylation, changes in Aβ, β-secretase, reactive oxygen species, tumor necrosis factor-α, cyclooxygenase-2, and interleukin-1β production, and promotion of Aβ25–35 disaggregation). The most effective of these compounds enhances learning, memory, and novel object recognition in mice, with good BBB permeability [135].

#### 3.5.28. Doxycycline

Doxycycline, a second-generation tetracycline antibiotic, has been proposed as a candidate MT drug for AD. Doxycycline has anti-AβOs, anti-inflammatory activities, good BBB penetration, and a safe pharmacological profile [136].

#### 3.5.29. Tryptanthrin Derivatives with Benzenesulfonamide Substituents

Two series of tryptanthrin derivatives with benzenesulfonamide substituents exhibit potent cholinesterase inhibition and neuroprotective properties. One derivative in particular acts as a mixed reversible dual inhibitor of AChE and BuChE, preventing the formation of amyloid plaques by inhibiting self-induced Aβ aggregation. It also exerts anti-neuroinflammatory effects on nitric oxide (NO), interleukin-1β (IL-1β), and tumor necrosis factor-α (TNF-α), reduces reactive oxygen species (ROS) production, and chelates biometals. This compound also ameliorates learning and memory deficits in a scopolamine-induced AD mouse model [137].

#### 3.5.30. Dimethyl Fumarate Plus Tranilast-Modified Dithiocarbate

The combination of the pharmacophoric features of Dimethyl fumarate, Tranilast, and Dithiocarbate generates a series of MT molecules that inhibit AChE, activate Nrf2, promote the nuclear translocation of Nrf2 protein, and induce the expressions of Nrf2-dependent enzymes, HO-1, NQO1, and GPX4. Some of these compounds also rescue BV-2 cells from H_2_O_2_-induced injury and inhibit ROS accumulation. Their anti-neuroinflammatory effect is related to a reduction in the levels of the pro-inflammatory cytokines NO, IL-6, and TNF-α, as well as suppression of the expressions of iNOS and COX-2. In addition, they improve memory impairment in a Scopolamine-induced mouse model [138].

#### 3.5.31. Polysubstituted Pyrazine Derivatives

Compound **A3B3C1** is a polysubstituted pyrazine derivative with MT activity. A3B3C1 acts on the Nrf2/ARE signaling pathway, increases the expression of NQO1 and HO-1, affects Aβ1-42 self-aggregation and deaggregation, inhibits BACE-1, promotes metal chelation, and protects SH-SY5Y cells against H_2_O_2_-induced oxidative damage [139].

#### 3.5.32. Thiosemicarbazones

Thiosemicarbazones, derived from the donepezil pharmacophore, 1-benzylpiperidine, may target 5 AD phenotypic features (cholinergic deficit, oxidative stress, dysfunctional autophagy, metal dyshomeostasis, protein aggregation). Pyridoxal 4-N-(1-benzylpiperidin-4-yl)thiosemicarbazone (PBPT), the lead compound in this series, affects iron chelation, oxidative stress, AChE activity, induction of autophagy, and inhibition of copper-mediated Aβ aggregation [140].

#### 3.5.33. PhenylSulfonyl–Pyrimidine Carboxylate Derivatives

Phenylsulfonyl–pyrimidine carboxylate (BS-1 to BS-24) derivatives inhibit AChE, BuChE, and self-induced and AChE-induced Aβ1-42 aggregation [141].

#### 3.5.34. Protriptyline

Among 140 FDA-approved CNS drugs, protriptyline shows MT properties as an inhibitor of AChE, β-secretase (BACE-1), and glycation-induced Aβ aggregation [142].

#### 3.5.35. Amiridine Hybrids

Amiridine is a cholinesterase inhibitor. When amiridine is used as a core structure and paired with memantine/adamantylamine, trolox, and substituted benzothiazole moieties, it generates a novel compound with NMDA receptor affinity, antioxidant capacity, BuChE inhibition, and anti-amyloid properties [143].

#### 3.5.36. Phosphodiesterase 2 Inhibitors

Phosphodiesterase-2 (PDE2) is a target for AD. Some novel PDE2 inhibitors show potential MT effects with antioxidant capacities and inhibitory activity against PDE2A [144].

#### 3.5.37. 2-Arylbenzofuran Derivatives

Some compounds of a new series of 2-arylbenzofuran derivatives act as dual cholinesterase inhibitors, comparable to donepezil, and as β-secretase inhibitors, with superior efficacy to baicalein [145].

#### 3.5.38. Salicyladimine Derivatives

Salicyladimine derivatives are MT agents in AD models with inhibitory activity against Aβ aggregation and MAO-B, antioxidant effects, metal chelation, and good BBB penetration. Some of these compounds exhibit neuroprotective effects by reducing ROS generation, protecting against H_2_O_2_-induced apoptosis, preventing 6-OHDA-induced cell injury and having anti-inflammatory activity [146].

#### 3.5.39. 3-Arylbenzofuranone Derivatives

MT 3-arylbenzofuranone derivatives show anti-ChE, anti-MAO, and antioxidant activities [147].

#### 3.5.40. CNI-1493 and C1213

CNI-1493 is a drug with anti-inflammatory properties and neuroprotective capacity in CRND8 transgenic AD mice. C1213 is an analog of CNI-1493 without anti-inflammatory effects. Both compounds interact with Aβ aggregates and attenuate Aβ cytotoxicity. CNI-1493 and C1213 ameliorate Aβ-induced behavioral deficits in nematodes and reduce Aβ plaque burden and cognitive deficits in transgenic CRND8 mice [148].

#### 3.5.41. Fluoren-9-Amines

Novel fluoren-9-amine derivatives are selective BuChEIs and antagonists of NMDA receptors at the GluN1/GluN2A and GluN1/GluN2B subunits [149].

#### 3.5.42. Flavone–Cyanoacetamide Hybrids

Within a series of 19 flavone–cyanoacetamide hybrids, some compounds are AChEIs, with antioxidant activity, anti-self-induced Aβ aggregation, and neuroprotection in SK-N-SH human neuroblastoma cells [150].

#### 3.5.43. Indazole Ethers

Some new indazole ethers are BuChEIs and agonists of cannabinoid CB2 [151].

#### 3.5.44. Hyaluronan–Carnosine Conjugates

New hyaluronic acid derivatives (200 and 700 kDa) conjugated with carnosine (Car) show Aβ antiaggregant ability. These bioactive HyCars inhibit the formation of Aβ42 aggregates and dissolve amyloid fibrils to reduce Aβ-induced toxicity in vitro [152].

#### 3.5.45. Melatonin-Derived Benzylpyridinium Bromides

Compounds from a series of melatonin-derived benzylpyridinium bromides show cholinesterase inhibition, antioxidant properties, and neuroprotective effects [153].

#### 3.5.46. Toluidine Blue O

Toluidine blue O (TBO) is a cholinesterase inhibitor that affects APP processing, tau phosphorylation, and tau kinase/phosphatase activity in N2a mouse neuroblastoma cells expressing the APP695 (N2a-*APP*Swe) Swedish mutation. TBO decreases the expression of *BACE1*, *PS1*, and *APP* and reduces the levels of Aβ40/42. The levels of tau and phosphorylated tau, at residues Ser202/Thr205, Thr181, Ser396, and Ser396/Ser404, are also reduced by TBO due to downregulating the expression of Akt, GSK-3β, Cdk5, and inactive p-PP2A while upregulating the expression of p-Akt (Ser473) and inactive p-GSK-3β (Ser9) [154].

#### 3.5.47. Pyrimidine/Pyrrolidine–Sertraline Based Hybrids

Some Pyrimidine/Pyrrolidine–Sertraline hybrids are potent inhibitors of AChE, BuChE, MAO-A, MAO-B, and BACE-1 [155].

#### 3.5.48. Quinazolinone Derivatives

Novel quinazolinone-based derivatives show MT anti-AD activity, exhibiting cholinesterase inhibitory and anti-inflammatory properties, with additional effects on hippocampal inflammatory markers (TNF-α, NFĸB, IL-1β, IL-6), antioxidant markers (SOD), and neuroprotection [156].

#### 3.5.49. Histamine H3 Receptor Ligands

Xanthone derivatives targeting histamine H3 receptors (H3R), as antagonists/inverse agonists, show affinity for hH3R, with cholinesterase and MAO-B inhibitory activity. Two azepane derivatives, 23 (2-(5-(azepan-1-yl)pentyloxy)-9H-xanthen-9-one) and 25 (2-(5-(azepan-1-yl)pentyloxy)-7-chloro-9H-xanthen-9-one), exhibit high affinity for hH3R, strong inhibitory activity against AChE, and moderate inhibitory activity against BuChE and hMAO-B. These compounds also showed memory-enhancing effects and favorable analgesic properties [157].

Among non-imidazole histamine H3 receptor ligands, 1-[2-thiazol-5-yl-(2-aminoethyl)]-4-n-propylpiperazine, 1-[2-thiazol-4-yl-(2-aminoethyl)]-4-n-propylpiperazine, and 1-phenoxyalkyl-4-(amino)alkylopiperazine exhibit MT properties. Methyl(4-phenylbutyl){2-[2-(4-propylpiperazin-1-yl)-1,3-thiazol-5-yl]ethyl}amine (A12) is a promising compound with H3 antagonist activity, inhibitory effects on AChE and BuChE, and anti-amnestic properties [157,158]. Novel **guanidines** (ADS10310), with a histamine H3R antagonist/inverse agonist profile, show moderate inhibition of BuChE and AChE [159]. Alicyclic amines linked by an alkoxy bridge to an aromatic lipophilic moiety of [1,1′-biphenyl]-4-carbonitrile represent a new series of cyanobiphenyls with MT properties in AD models. These compounds show high affinity for human histamine H3 receptors (hH3R) and nonselective inhibitory activity against AChE, BuChE, and MAO-B. One of these multiple hH3R/eeAChE/eqBuChE/hMAO B ligands shows good metabolic stability, moderate hepatotoxicity, and a beneficial effect on scopolamine-induced memory impairments [160].

#### 3.5.50. Alkyl-Substituted 4-Methoxy Benzaldehyde Thiosemicarbazones

Novel thiosemicarbazones show anti-AChE activity and inhibition of the GluN1-1a + GluN2B subunit of N-methyl-D-aspartate receptors. Pre-treatment of BV-2 microglial cells with one of these compounds effectively decreases nitrite production. They also regulate autophagy in transfected SH-SY5Y neuroblastoma cells and decrease copper-catalyzed oxidation of Aβ [161].

#### 3.5.51. Biomimetic Dendrimer–Peptide Conjugates

A ROS-responsive dendrimer–peptide nanoconjugate (APBP) may contribute to restoring the antioxidant ability of neurons by activating the nuclear factor (erythroid-derived 2)-like 2 signaling pathway. This nanoconjugate reduces ROS levels, decreases Aβ burden, alleviates glial cell activation, and enhances cognitive functions in APPswe/PSEN1dE9 transgenic mice [162].

#### 3.5.52. MT Anti-Neuroinflammatory Agents

Daniela Melchiorri and colleagues screened 2226 clinical trial records and 20 patents for drugs with anti-neuroinflammatory profiles and found agents that target intracellular inflammatory kinase signaling (Baricitinib, NE3107, MW150, Neflamapimod/VX-745), inhibitors of cytokines or eicosanoids (XPro1595/Pegipanermin, Canakinumab, Lenalidomide, Emtricitabine, Montelukast, Salsalate, ALZT-OP1), modulators of microglia and astrocytes activation (AL002, TB006, Edicotinib, Sargramostim, Pepinemab, Daratumumab), and immunomodulatory agents (VT301/GB301) with potential MT activity [163]. Cathepsin B (CatB), dual specificity phosphatase 2 (DUSP2), and monoglycerol lipase (MAGL) are targets for multi-target-directed ligand (MTDL) drug development in AD. Inhibitors of CatB, DUSP2, and MAGL have the capacity to slow the neuroinflammatory component of AD [164].

### 3.6. Anti-Amyloid Agents

Many experimental drugs targeting Aβ pathology demonstrated potential utility in preclinical studies; however, the vast majority of them have not been able to reduce the progress of AD and memory impairment in clinical studies [165]. Despite all this, the presence of Aβ deposits remains the main hallmark of AD, and the elimination of Aβ from the brain is the first therapeutic objective contemplated in almost all MT strategies [166].

The pharmaceutical categories of the various anti-amyloid strategies (some of which have potential MT profiles) tested in recent years are as follows: (i) Secretase modulators: β-secretase inhibitors (BACE1) (Verubecestat/MK-8931, Lanabecestat/AZD3293, Atabecestat/JNJ-54861911, LY3202626, Umibecestat/CNP520, Elenbecestat/E2609); Inhibitors and modulators of γ-secretase (Semagacestat/LY450139, Avagecestat, Tarenflurbil/R-flurbiprofen, EVP-0962/EVP-0015962, Itanapraced/CHF5074, BMS-932481, Pinitol/NIC5–15); α-secretase activators (Etazolate/EHT-0202, Acitretin, Epigallocatechin-3-gallate, Bryostatin 1, APH-1105, ID1201). (ii) Specific anti-Aβ immunotherapy: Active anti-amyloid immunotherapy (AN1792, Affitope AD02, CAD106/Amilomotide, ACC-001/Vanutide cridificar, ACI-24, Lu AF20513, ABvac40, UB-311, NPT088/M13F, AV-1959D); first-generation passive anti-amyloid immunotherapy (Bapineuzumab/AAB-001, Crenezumab/MABT5102A/MABT, Ponezumab/PF-04360365, Gantenerumab, Solanezumab, Solanezumab + Gantenerumab); second-generation passive anti-amyloid immunotherapy (Aducanumab/BiiB037, Lecanemab/BAN2401/mAb158, Donanemab/LY3002813, 72D9, ACU-193, H3D6 antibody, 11E12 antibody). (iii) Compounds for the elimination of amyloid aggregates and deposits: Compounds for activation of the enzymes responsible for amyloid plaque degradation (neprilysin, insulin-degrading enzyme (IDE), plasmin, endothelin-converting enzyme, angiotensin-converting enzyme (ACE), Cathepsin D, metalloproteinase 9). (iv) Modulators of Aβ clearance/aggregation (APOE, LRP (low-density lipoprotein receptor-related protein), receptor for advanced glycation end products (RAGE), ApoJ, α2-Macroglobulin (α2M); Azeliragon/PF-04494700/TTP4000 (RAGE inhibitor); Scyllo-inositol/ELND005; ALZT-OP1 (cromolyn) + ALZT-OP2 (ibuprofen); GV-971 (Sodium oligomannate); ALZ-801); Small molecules to target signal transduction pathways activated by toxic Aβ aggregates (Elayta/CT1812, sigma2 receptor antagonist; Saracatinib/AZD0530, dual inhibitor of the tyrosine kinases c-Src and Abl) [165].

### 3.7. Potential Anti-Tauopathic Drugs

The tauopathic component of AD is represented by intracellular neurofibrillary tangles (NFTs) formed due to hyperphosphorylation of the tau protein. The six isoforms of tau (encoded in a 16-exon gene on chromosome 17q21) are post-translationally modified by phosphorylation, acetylation, ubiquitination, SUMOylation, glycosylation, nitration, methylation, prolyl-isomerization, glycation, and truncation. The primary function of tau is microtubule assembly and regulation of intracellular trafficking.

The pathogenic mechanisms of tau in AD are associated with tau post-translational modifications, especially hyperphosphorylation, and also SUMOylation, nitration, glycation, methylation, prolyl-isomerization, and aggregation, a mixture of 3- and 4-repeat tau isoforms (3R and 4R) in assembled states that contain paired helical filaments (PHFs), straight filaments and oligomers [167] that contribute to neuronal death. The amount of NFTs correlates with the degree of dementia [168].

Small molecules targeting tau in AD models include tau post-translational modification modulators, aggregation inhibitors, and degradation promotors [169].

#### 3.7.1. Modulators of Tau Post-Translational Modifications

Agents modulating tau post-translational modifications can be classified into several categories: (i) Modulators of tau phosphorylation: (a) Phosphatase activators (Sodium selenate (Na2SeO4), a negatively charged anionic compound that activates PP2A (Phosphoprotein phosphatase 2A); Memantine; ApoE mimetic, COG112 (acetyl-RQIKIWFQNRRMKWKKCLRVRLASHLRKLRKRLL-amide), also a PP2A activator; Fingolimod, a sphingosine-1-phosphate receptor agonist; SEW2871, a selective sphingosine-1-phosphate receptor agonist; Genistein; Metformin; Resveratrol; Cornel iridoid glycoside (Morroniside and Loganin, increase PP2A activity via inhibiting PP2Ac demethylation resulting in inhibition of tau hyperphosphorylation); Allosteric activators of PP2A (Ceramide analogs: Sphingosine, Sphingosine phosphate, Ceramide phosphate, 1-O-Methyl-C6-ceramide, and other PPA2 activators (Dithiolethione, Xylulose-5-phosphate, Eicosanoyl-5-hydroxytryptamide, Taurolidine, 1,8-Naphthyridines); (b) Kinase inhibitors (proline-directed kinase, non-proline-directed kinase, and tyrosine protein kinases) (glycogen synthase kinases 3 alpha and beta (GSK3α, GSK3β) (GSK3β inhibitors: Tideglusib, Lithium), cyclin dependent kinase 5 (CDK5), mitogen-activated protein kinase family (MAPKs), leucine-rich repeat kinase 2 (LRRK2), Akt (protein kinase B), c-Abelson (c-Abl), dual-specificity tyrosine phosphorylation-regulated kinases (DYRK1A) and Fyn (Fyn inhibitor: Saracatinib), all involved in tau phosphorylation. (ii) Modulators of tau acetylation: (a) Acetylation inhibitors: Salsalate, a prodrug of salicylate with non-steroid anti-inflammatory effects, is an inhibitor of acetyltransferase p300-induced tau acetylation [170]; C646, a pyrazolone-containing small-molecule inhibitor of p300; CGP3466B (omigapil), a glyceraldehyde-3-phosphate dehydrogenase nitrosylation inhibitor; Sirtuin modulators; (b) Histone deacetylase (HDAC) inhibitors: ACY-738 (HDAC6 inhibitor); CKD-504 (HDAC6 inhibitor); Glycodeoxycholic acid (HDAC6 inhibitor); RGFP-966 (HDAC3 inhibitor). (iii) Modulators of tau glycosylation (O-GlcNAcase inhibitors): PUGNAc, NAG-thiazoline, NButGT, Thiamet-G, MK-8719, ASN120290 (ASN-561). (iv) Modulators of tau truncation (caspase inhibitors): Benzyloxycarbonyl-valine-alanine-aspartate-fluoromethylketone (Z-VAD-FMK), Quinolyl-valyl-O-methylaspartyl-(-2, 6-difluorophenoxy)-methyl ketone (Q-VD-OPh), Minocycline [169].

#### 3.7.2. Tau Aggregation Inhibitors

Small molecules capable of inhibiting tau aggregation include methylene blue, LMTM (TRx0237), curcumin derivatives (PE859, (3-[(1E)-2-(1H-indol-6-yl)ethenyl]-5-[(1E)-2-[2-methoxy-4-(2-pyridylmethoxy) phenyl] ethenyl]-1H-pyrazole, curcumin-derived pyrazoles and isoxazoles), N744, rhodanines (Compound RH-1), aminothienopyridazines (ATPZs), cinnamaldehyde, epicatechin, crocin, VB-003 (GSK3β inhibitor), VB-008, AM404 (anandamide transport inhibitor), and N-(3-chloro-1,4-dihydro-1,4-dioxo-2-naphthalenyl)- l-tryptophan (Cl-NQTrp) [169].

#### 3.7.3. Promoters of Tau Degradation

The ubiquitin–proteasome system (UPS) and the autophagy-lysosome pathway (ALP) are the two main protein degradation pathways in neurons, which are compromised in tauopathies. The stimulation of the proteasome-mediated proteolysis, the inhibition of ubiquitin-specific peptidase 14 (USP14), the enhancement of cAMP or cGMP signaling systems by phosphodiesterase (PDEs) inhibitors, and the increase in the recruitment of ubiquitin machinery to tau via proteolysis targeting chimeric molecules (PROTACs) may accelerate the rate of tau degradation [169]. (i) UPS activators: Proteasome stimulators (chlorpromazine, TCH-165); USP14 inhibitors (selective inhibitors of USP14, IU1 and IU1-47); PDEs inhibitors (Rolipram, PDE4 inhibitor; BPN14770, PDE-4D inhibitor; Cilostazol, PDE3 inhibitor; Sildenafil, PDE5 inhibitor); PROTACs (PROTAC QC-01-175). (ii) Autophagy activators: Targeting the mTOR-dependent pathways (rapamycin, temsirolimus); Targeting the mTOR-independent pathways (Trehalose, lithium chloride, Methylene blue, Nilotinib (a second-generation Abelson (Abl) tyrosine kinase inhibitor), Pimozide, lonafarnib (a farnesyltransferase inhibitor). (iii) Chaperone and co-chaperone modulators: Hsp70 inhibitors (phenothiazines (methylene blue and azure C), flavones (myricetin) and rhodacyanines (MKT-077, YM-01, YM-08, JG-48, and JG-98)); Hsp90 inhibitors (geldanamycin analogs (17-AAG), purine class EC102, PU-DZ8, and PU24FCl, dihydropyridine derivatives (LA1011), novobiocin analogs (KU32), celastrol); Co-chaperones modulators (sulforaphane, KU-177, Curcumin) [169].

#### 3.7.4. Potential MT Anti-Tau Drugs

Although many small molecules with anti-Tau effects have been investigated, none have been sufficiently effective in neutralizing the complex pathogenesis of AD. The search for MT drugs with anti-Tau action simultaneously on other pathogenic targets has been equally unsuccessful so far. However, there are some examples of potential anti-tau MT agents: Shogaol–huprine hybrid, levetiracetam–huprine hybrid, heptamethylene-linked levetiracetam–huprine, levetiracetam–(6-chloro)tacrine, and LM-031 [169].

### 3.8. Natural Bioproducts

Many natural products exhibit an MT profile with beneficial effects in AD models [171,172,173,174]. There are multiple classifications of natural bioproducts with potential MT activity in AD models. A recent classification organizes these bioproducts into the following categories: (i) Phenylpropanoids: ferulic acid derivatives, coumarin derivatives, eugenol derivatives; (ii) Flavonoids derivatives: quercetin and kampferol derivatives, liquiritigenin and isoliquiritigenin derivatives, chalcone derivatives; (iii) Terpenoids derivatives: triptolide derivatives, andrographolide derivatives, glycyrrhetinic acid derivatives, safranal derivatives; (iv) Saponin derivatives: diosgenin derivatives, sarsasapogenin derivatives; (v) Alkaloids derivatives: piperine derivatives, harmine derivatives, oxymatrine derivatives [171].

The number of natural bioproducts studied over the last 20 years is impressive, with evidence of potential MT effects in AD models. Table S5 lists the most significant bioproducts with documented MT activity in alphabetical order. Most of these products originate from traditional Oriental medicine practices in China, Korea, Japan, and Ayurvedic medicine [175,176,177,178,179,180,181,182]. Notable, as well, is the increasing number of marine bioproducts investigated for potential MT effects in AD models [183] (Table S5).

### 3.9. Epigenetic Drugs

An increasing number of studies show the pathogenic role of various epigenetic aberrations in AD [12,184,185,186,187]. Changes in DNA methylation, chromatin and histone modifications, and dysregulation of microRNAs are essential mechanisms in gene expression regulation. These epigenetic mechanisms are becoming attractive molecular targets for developing therapeutic approaches to AD and optimizing the personalization of pharmacological treatments for dementia through pharmacoepigenetic procedures [188,189].

Although pharmacoepigenetics and the development of epigenetic drugs (epidrugs) for AD treatment are in a very preliminary phase, progress is being made in developing epidrugs for AD [189]. Both novel and repurposed drugs with epigenetic effects are in clinical trials to test their potential effects on AD [190]. Furthermore, the first pharmacoepigenetic studies performed with MT treatments based on a multifactorial strategy demonstrate that *SIRT2*, *APOE*, and *CYPD6* are genes that cooperate in the efficacy and safety of anti-AD treatments [191].

Epigenetic drugs (Tables S3 and S6), which target modifications that regulate gene expression without altering the DNA sequence, modify the epigenetic landscape that contributes to the pathogenesis of AD, including the expression of genes involved in amyloid-beta (Aβ) production, tau hyperphosphorylation, neuroinflammation, and synaptic function [188,189].

Key epidrugs for AD include (i) **HDAC Inhibitors**. HDAC inhibitors (Vorinostat, Tucidinostat, Romidepsin, Panobinostat, Belinostat) prevent the removal of acetyl groups from histone proteins, leading to a more relaxed chromatin structure and increased gene expression. In AD, they are believed to enhance the expression of neuroprotective genes and improve synaptic plasticity. Some HDACs are altered in AD and contribute to AD pathogenesis. HDAC inhibitors are being investigated as potential MT drugs for AD treatment with unsuccessful results [189,192]. **Vorinostat (SAHA)** has shown potential in preclinical models of AD by enhancing memory and reducing neurodegeneration. **Valproic acid**, an anticonvulsant with HDAC inhibitory activity, has been studied for its ability to reduce Aβ production, although results in clinical trials have been mixed. The pan-HDAC inhibitor **sodium valproate** (VPA) and the HDAC6 selective inhibitor **WT161** may show some beneficial effects in AD. VPA and WT161 downregulate the expression of multiple HDACs and affect the expression of APP and APP secretases (BACE1, PSEN1, ADAM10), reducing Aβ deposition in AD cell and mouse models [193]. **Sodium Butyrate**, a short-chain fatty acid with HDAC inhibitory effects, improves cognitive function and reduces Aβ and tau pathology in animal models. Most of the FDA-approved epidrugs are used in the treatment of different forms of cancer. Currently, several HDAC inhibitors (Abexinostat, Fimepinostat (CUDC-907), Quisinostat (JNJ26481585), Ricolinostat (ACY-1215), Trichostatin A, Nanatinostat (VRx-3996), CG200745, Pracinostat, Resminostat, CUDC-101, MPT0E028) are in different phases of clinical trials, most of them with anti-cancer objectives. AMX0035, a carboxylic acid Pan-HDAC, is in Phase 2/3 clinical trials for AD and amyotrophic lateral sclerosis [194]. (ii) **DNA Methyltransferase (DNMT) Inhibitors.** DNMT inhibitors prevent the addition of methyl groups to DNA, which can reactivate silenced genes, including those involved in neuroprotection and synaptic function. **Azacytidine** and **Decitabine** are nucleoside analogs that incorporate into DNA and inhibit DNMTs, leading to DNA hypomethylation. While primarily used in cancer treatment, their potential to reactivate silenced neuroprotective genes is being explored in AD research. (iii) **Bromodomain and Extra-Terminal Domain (BET) Inhibitors.** BET inhibitors target proteins that recognize acetylated lysines on histones, affecting gene expression. They can modulate inflammation and have shown promise in preclinical models of neurodegenerative diseases. JQ1 is a BET inhibitor that has shown neuroprotective effects in AD models by reducing neuroinflammation and improving cognitive function. (iv) **Sirtuin Activators.** Sirtuins are a class of NAD^+^-dependent deacetylases that play a role in cellular aging and metabolism. Activating sirtuins, particularly SIRT1, is neuroprotective. **Resveratrol** activates SIRT1 and has been studied for its potential to protect against neurodegeneration and improve cognitive function in AD. **Nicotinamide Riboside**, a precursor to NAD^+^, boosts SIRT1 activity and shows potential in preclinical studies for improving mitochondrial function and reducing neuroinflammation in AD. (v) **Histone Acetyltransferase (HAT) Activators.** HAT activators enhance the addition of acetyl groups to histones, promoting gene expression. These drugs aim to counteract the epigenetic repression observed in AD. **Curcumin** is a natural HAT activator with neuroprotective and anti-inflammatory effects in AD [12,189,195].

Histone lysine-specific demethylase 1 (LSD1/KDM1A) is an epigenetic enzyme that demethylates specific lysine residues of histone H3 (H3K4me1/2 and H3K9me1/2). LSD1 is involved in the pathogenesis of cancer. Covalent and non-covalent **LSD1 inhibitors** have been studied in clinical trials for the treatment of hematological disorders and solid cancers. Some of them (tranylcypromine, iadademstat (ORY-1001), bomedemstat (IMG-7289), GSK-2879552, INCB059872, JBI-802, and Phenelzine) covalently bind the FAD cofactor. Pulrodemstat (CC-90011) and Seclidemstat (SP-2577)] are non-covalent LSD1 inhibitors. Vafidemstat (ORY-2001), a tranylcypromine-based LSD1/MAO-B dual inhibitor, is in clinical trials for treating AD and personality disorders [196].

**Smilagenin** (SMI), a lipid-soluble steroidal sapogenin from the traditional Chinese medicinal herb *Radix Asparagi*, extracted from *Asparagus cochinchinensis* (Lour.) Merr., increases brain-derived neurotrophic factor (BDNF) expression in SH-SY5Y cells treated with Aβ. SMI enhances *BDNF* mRNA expression, global H3AC and H4AC levels, and *P300* expression in AD models. SMI epigenetically regulates *BDNF* expression through HAT enhancement. SMI upregulates histone acetylation of BDNF, and SMI-related cognitive improvement is abolished following P300 inhibition in *APP*/*PS1* mice [197].

**MicroRNAs.** MicroRNAs (miRNAs) control gene expression and regulate neurogenesis, neurodifferentiation, dendritic sprouting, and synaptogenesis in the CNS. The expression levels of miRNAs are altered in AD, contributing to AD pathogenesis. Targeting disrupted miRNAs may represent a novel MT approach against AD [198].

One of the main challenges in developing epigenetic drugs for AD is achieving target specificity. Broad-spectrum inhibitors, such as HDAC inhibitors, can affect a wide range of genes, potentially leading to unintended side effects. Although many epigenetic drugs show promise in preclinical models, translating these findings into effective treatments for Alzheimer’s patients has proven challenging. Given the multifactorial nature of AD, epigenetic drugs may be most effective when used in combination with other therapeutic strategies, such as anti-amyloid and anti-tau therapies.

## 4. Nosustrophine: A Prototype of Epipleiotropic Agent for AD Prevention and Treatment

Nosustrophine (NST) is a bioproduct obtained from the juvenile brain of *Sus scrofa domestica* by a non-denaturing biotechnological process (Patent ID: P202230047/ES2547.5). The ultrapure freeze-dried extract of NST contains, among other complex brain components, fatty acids of the omega-3, -6, and -9 types (saturated: stearic acid, 28%; palmitic acid, 11%; monosaturated: oleic acid, 28.1%; polysaturated: docosahexaenoic acid, 14%; arachidonic acid, 13.6%), with no trans fatty acids; 18 amino acids (glutamic acid, 6%; aspartic acid, 5.1%; leucine, 4.4%; arginine, 3.7%; lysine, 3.4%; serine, 3.4%; alanine, 2.9%; glycine, 2.7%; phenylalanine, 2.6%; proline, 2.3%; valine, 2.2%; 2.1%; tyrosine, 2.1%; isoleucine, 1.7%; cysteine, 0.73%; methionine, 0.6%; tryptophan, 0.55%); vitamins (riboflavin (B2), 82 mg/Kg; niacin (B3), 17 mg/Kg; cholecalciferol (D3), 8.1 mg/Kg; tocopherol (E), 184 mg/Kg); eight minerals (calcium, 503 mg/Kg; magnesium, 93 mg/Kg; zinc, 60 mg/Kg; iron, 52 mg/Kg; copper, 17 mg/Kg; potassium, 13 mg/Kg; manganese, 4 mg/Kg; selenium, 1.06 mg/Kg), and several brain regulatory elements, including synthesis precursors (L-DOPA, 22.5 mg/g), neurotransmitters (dopamine, 2760 pg/mg; noradrenaline, 655 pg/mg; serotonin, 479 pg/mg; histamine, 158 pg/mg), neuropeptides (corticotropin-releasing hormone, 0.658 pg/mg; somatostatin, 7.75 pg/mg) and neurotrophic factors (brain-derived neurotrophic factor, 35 pg/mg) [199].

The proteomic analysis of NST with LC-MS/MS identified a total of 517 proteins, including proteins that are linked to AD, such as Aβ A4 [200], adenosylhomocysteinase (AHCY) [201]; apolipoproteins E, A, and C [9,202]; cathepsin D [203]; choline O-acetyltransferase [3,204]; neuroendocrine protein 7B2 [205]; nicotinamide phosphoribosyltransferase (NAMPT) [206]; and presenilin 2 [199,207]. Other proteins involved in AD-related pathogenic pathways (i.e., neurotrophic function, neurotransmitter metabolism, neuroinflammation, oxidative stress, brain oxygenation, cellular apoptosis, synaptic function, and brain metabolism) are also present in the complex structure of NST [199].

### 4.1. Neuroprotective, Antioxidant, Anti-Inflammatory, Anti-Amyloid, and Neurotrophic Effects

In cell culture models, NST improves cell viability of SH-SY5Y neuroblastoma and HepG2 hepatocarcinoma cell lines, sustains neuronal survivability rates, enhances glial cell survival in mouse primary neuronal and glial cells, and is devoid of toxic effects on human cell lines. The protective effect of NST on microglia inversely correlates with the drug concentration in the culture medium. NST is neuroprotective against Aβ1-42-induced neurodegeneration in mouse organotypic hippocampal slice cultures [208].

In experimental *3xTg-APP/Bin1/COPS5* transgenic mice models of AD, NST enhances the activity of the immune system and reduces pathological changes in the hippocampus and cortex by halting the development of amyloid plaques, more significantly in young transgenic mice, indicating that NST effects are more relevant in early stages of the disease, which gives NST the character of a preventive agent [209].

In 3xTg-AD mice, NST reduces the concentration of reactive C protein (RCP) (a biomarker of inflammation), increases total antioxidant capacity (TAS) and glutathione reductase (GR) activity, an antioxidant enzyme, and decreases creatinine concentration and liver enzyme activity (GOT and GPT transaminases) [209].

**Figure 3:**
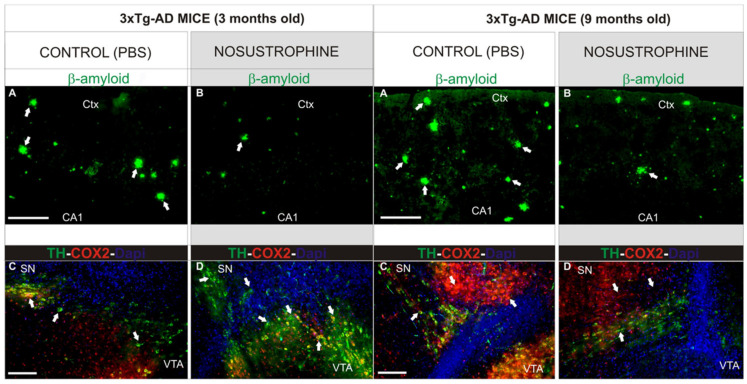
Effect of Nosustrophine on neuropathological hallmarks in transgenic AD mouse models. Comparative photomicrographs of 3xTg-AD mouse brain sections from 3- and 9-month-old mice, representing early and late stages of AD neuropathology, stained with anti-Aβ (**A**,**B**), anti-TH, and anti-Cox2 antibodies (**C**,**D**). Images of the cortical areas demonstrate how 3xTg-AD mice treated with Nosustrophine (**B**) exhibit a significant reduction in Aβ plaques compared to control (untreated) mice (**A**). Treatment with Nosustrophine (**D**) also significantly attenuates the inflammatory reaction (COX-2) present in dopaminergic neurons (TH) of the midbrain of untreated transgenic animals (**C**). Scale bar: 100 μm.

Amyloid plaque burden, astrogliosis (GFAP), immune activation (IL-17, CD11b), apoptosis induction (Cox-2), neuronal differentiation (TH), and development (NeuN) are highly influenced by NST in 3xTg-AD mice. NST displays neurotrophic effects, which induce neuronal development in areas of neurogenesis (lateral ventricles and the dentate gyrus of the hippocampus) and reduces the density of amyloid plaques in the ventricular ependyma, which is strongly affected during the neuropathological development of the disease. NST reduces neurodegeneration by maintaining neuronal density in the regions affected by Aβ plaques, especially in the neocortex and CA1 region of the hippocampus (Figure 3). NST-treated 3xTg-AD mice show a reduced amyloid deposition that is continuously sustained during development and significantly dimmer in density during the development of the neuropathological process of AD from 3 to 9 months compared to 3xTg-AA control mice. NST induces a 60% reduction in the density of Aβ plaques in 3xTg-AD animals [209] (Figure 3).

NST reduces inflammation in cortical and hippocampal regions of *Bin1*/*Cops5*/*App* transgenic mice. NST improves neuronal survival by inhibiting astrogliosis and reactive microglia activation in the neocortex and hippocampus. The high density of IL-17-positive cells in control animals, indicating extensive inflammation, is reduced by NST. The decrease in the density of catecholaminergic neurons in the midbrain, one of the main pathological hallmarks of AD in 3xTg-AD mice, is attenuated by treatment with NST, as reflected by Cox2-immunoreactivity, an indirect apoptotic marker (Cox2), proinflammatory cytokine (IL17), and a marker of dopaminergic neurons (TH). Brain function and psychomotor activity also improve in 3xTg-AD mice treated with NST [209].

### 4.2. Regulation of AD-Related Gene Expression

In 3- and 9-month-old *APP/BIN1/COPS5* triple-transgenic (3xTg) mice, which exhibit increased age-related Aβ accumulation and deposition and a gradual increase in cell death and neuroinflammation in the cortex and hippocampus [210,211], NST reduces the accumulation of Aβ by approximately 40%, as well as markers of neuronal death and neuroinflammation in the hippocampus, while increasing the levels of tyrosine hydroxylase (TH) in the ventral tegmental area of the midbrain, which are affected (71% lower in 3-month-old mice, reflecting an early pathogenic event compromising dopaminergic neurons) in this transgenic model [199]. VTA degeneration is one of the first events in the early (pre-plaque) stages of AD, with lower dopamine outflow towards the hippocampus, correlating with deficits in synaptic plasticity in the CA1 subfield and memory function [212,213,214].

**Figure 4:**
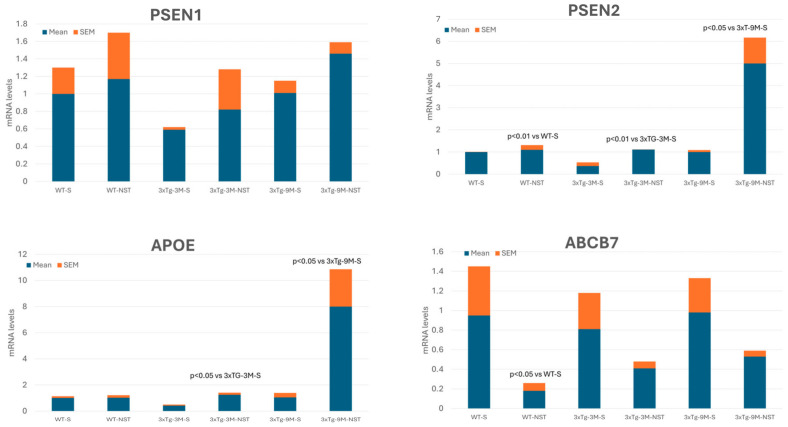
Regulation of AD-related gene expression by Nosustrophine (NST) in the hippocampus of 3- and 9-month-old wild type (WT) and AD-3xTg mice.

NST regulates the hippocampal expression of AD-related genes, such as presenilin 1 (*PSEN1*), presenilin 2 (*PSEN2*), apolipoprotein E (*APOE)*, microtubule-associated protein tau (*MAPT*), and ATP binding cassette subfamily B member 7 (*ABCB7*). NST does not affect *PSEN1* and *PSEN2* in wild-type animals; however, in *APP*/*BIN1*/*COPS5* mice, where the levels of *PSEN2* mRNA are 70% lower [211], NST increases *PSEN2* levels by 60%, reaching levels similar to wildtype animals [199] (Figure 4). 3xTg-AD mice have 65% lower *APOE* mRNA levels than wild-type mice [211], and NST causes three-fold-higher *APOE* levels in young *APP*/*BIN1*/*COPS5* mice, similar to wild-type animals [199]. NST does affect *MAPT* mRNA levels in this transgenic model. NST treatment decreases *ABCB7* expression in wild-type and 3xTg-AD mice (Figure 4). These findings clearly show that NST reduces mRNA levels of relevant AD-related genes in young animals and suggest that NST acts prophylactically when administered prior to the onset of AD pathology [199]. In 9-month-old *APP*/*BIN1*/*COPS5* mice, NST increases *PSEN2* and *APOE* expression five- and eight-fold, respectively, and decreases *ABCB7* expression, with a mild increase in *PSEN1* and *MAPT* expression levels (Figure 4). These findings indicate that NST regulates *PSEN2* and *APOE* gene expression in old 3xTg-AD mice when the neuropathological damage is fully consolidated, suggesting that NST possesses preventive and, to a lesser extent, therapeutic properties [199].

### 4.3. Regulation of Inflammation-Related Gene Expression

**Figure 5:**
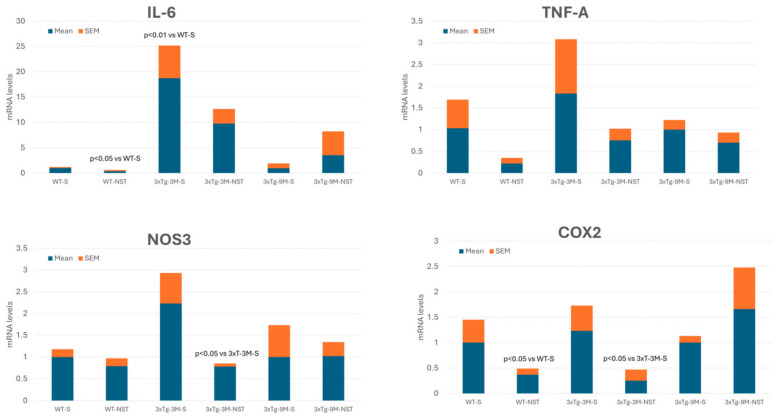
Nosustrophine (NST) regulation of inflammation-related gene expression in the hippocampus of 3- and 9-month-old wild type (WT) and AD-3xTg mice.

Neuro-inflammation influences AD onset and progression [215,216,217]. NST regulates the expression of some cytokines and inflammation-related genes in the *APP/BIN1/COPS5* transgenic model [199]. *APP*/*BIN1*/*COPS5* mice show higher *IL-6* and *TNFα* mRNA levels than wild-type mice. NST treatment reduces *IL-6* and *TNFα* levels in wild-type and *APP/BIN1/COPS5* mice (Figure 5).

Nitric oxide synthase 3 (NOS3) and cyclooxygenase-2 (COX-2) enzymes, linked to neuroinflammation [216,217], are upregulated in the *APP*/*BIN1*/*COPS5* mouse brain [44]. NST treatment reduces *NOS3* and *COX-2* expression to normal levels, indicating the NST decreases inflammation by regulating inflammation-related gene expression in young 3xTg-*APP*/*BIN1*/*COPS5* mice, with no effect on *IL-1β*, *IL-6*, *TNFα*, *COX-2,* and *NOS3* mRNA levels in older mice [199] (Figure 5). These findings appear to indicate that NST does not induce anti-neuroinflammatory effects against already established brain damage in AD pathology but is preventive against neuroinflammation in the early phases of the disease.

### 4.4. Epigenetic Effects

#### 4.4.1. DNA Methylation

**Figure 6:**
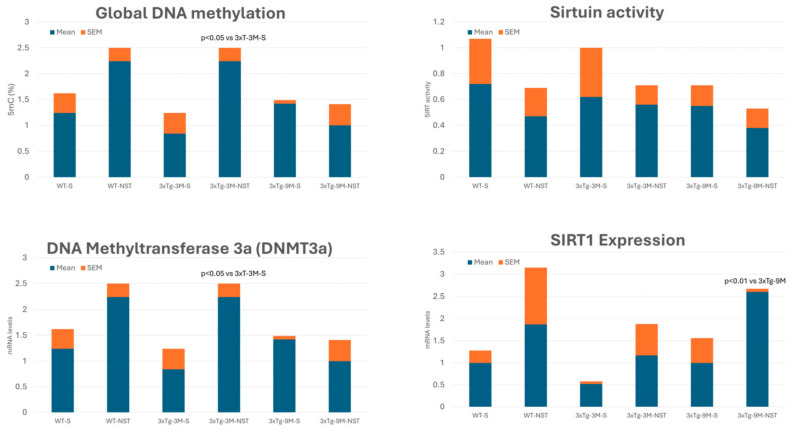
Nosustrophine regulation of DNA methylation, *DNMT3a* expression, SIRT activity, and *SIRT1* expression in the hippocampus of 3- and 9-month-old wild type and AD-3xTg mice.

Different epigenetic changes (DNA methylation, histone/chromatin modifications, microRNA dysfunction) are associated with AD neuropathology [189]. DNA methylation is a biomarker of AD [218,219]. NST treatment increases the levels of 5-methylcytosine (5mC) by 1.8-fold in the hippocampus of young wild-type mice and 2.8-fold in young *APP/BIN1/COPS5* animals. In contrast, NST does not affect DNA methyltransferase 1 (*DNMT1*) expression in wild-type and in *APP*/*BIN1*/*COPS5* mice. However, *DNMT3a* expression, which is reduced in the *APP*/*BIN1*/*COPS5* mouse brain, is increased more than three-fold versus young saline-treated *APP*/*BIN1*/*COPS5* mice, and this response is more significant in young than in old transgenic mice [199] (Figure 6). These findings indicate that NST has a strong effect on DNA methylation when taken as a preventive therapy, with reduced efficacy when AD-related neuropathology is already consolidated.

#### 4.4.2. Modulation of Sirtuin Activity

Sirtuins are implicated in various aging-related biological processes, such as oxidative stress and stress response, inflammation, mitochondrial dysfunction, and protein aggregation [220,221]. SIRT1 regulates APP processing, neurodegeneration, and neuroinflammation [221]. *SIRT2* mutations are associated with AD, and *SIRT2* variants affect the pharmacoepigenetic response to multifactorial treatments in AD [191]. *SIRT* mRNA and activity levels are lower in *APP*/*BIN1*/*COPS5* mice than in wild-type animals [211]. NST tends to increase *SIRT1* mRNA levels in wild-type and transgenic mice, but these differences are non-significant. However, in older 3xTg mice treated with NST, SIRT1 levels increase 2.5-fold, suggesting that NST upregulates *SIRT1* expression and promotes *SIRT1* transcription in 3xTg-AD mice [199] (Figure 6). NST-induced *SIRT1* mRNA upregulation has epigenetic effects in older 3xTg-AD mice by reducing (24%) a chromatin-associated substrate of SIRT1, acetylated-histone H3 (Lys14), in *APP*/*BIN1*/*COPS5* mice hippocampi [199].

#### 4.4.3. Regulation of Histone Deacetylase (HDAC) Activity

**Figure 7:**
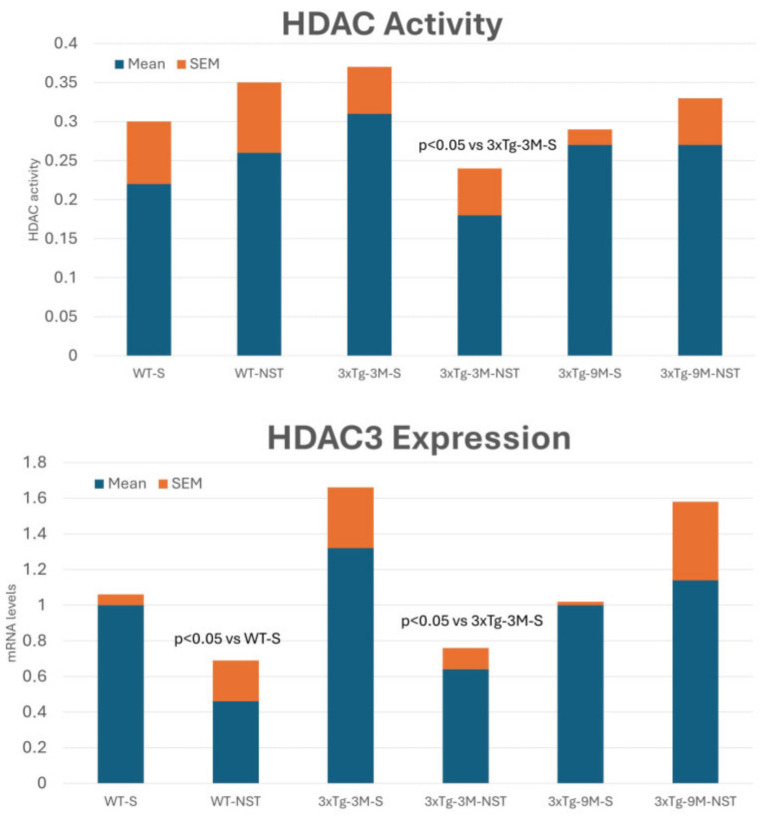
Effect of Nosustrophine on HDAC activity and *HDAC3* expression in the hippocampus of 3- and 9-month-old wild type and AD-3xTg mice.

Several lines of investigation show that some HDAC inhibitors are neuroprotective and increase synaptic plasticity, memory, and learning in AD models [191,222]. *APP*/*BIN1*/*COPS5* mice show higher HDAC activity and *HDAC3* expression than wild-type mice [211]. NST decreases (33%) HDAC activity and induces a two-fold reduction in *HDAC3* expression in the hippocampus of young transgenic mice, with no effect in older *APP*/*BIN1*/*COPS5* mice (Figure 7), suggesting that NST is a preventive treatment for AD by lowering HDAC activity and *HDAC3* expression [199].

## 5. Conclusions and Future Trends

Pharmacological research for the treatment of AD has been driven by trends in the last 50 years. The neuropeptides of the 1980s were followed in the 1990s by the fashion for AChEIs, and from the 2000s onwards came the fever of anti-amyloid vaccines. These trends have coincided with two key factors: first, novel discoveries regarding the pathogenesis of the disease, and second, pressure from certain organizations and the pharmaceutical industry on the scientific community and the FDA [1,2,44].

It is likely that the pathogenic complexity of AD requires a more complex intervention with novel chemicals, bioproducts, and/or MT drugs to neutralize all the endogenous and exogenous factors that contribute to the premature death of neurons. Likewise, there is clear evidence that the progressive and irreversible neurodegenerative process underlying the Alzheimer’s phenotype begins decades before the first symptoms appear [11]. Perhaps the onset of neurodegeneration is activated when the brain stops maturing between 30 and 40 years of age, at which time, a gene on/off switch phenomenon is likely to occur, with the participation of epigenetic changes that regulate gene expression and whose immediate consequence could be the beginning of latent neuronal destruction for decades [1,2,11]. The clinical onset of the disease represents a threshold of functional incapacity of the brain after the death of billions of neurons. Restoration with conventional treatments is not possible because no current treatment can resuscitate dead neurons or activate silent stem cells capable of replenishing dead neurons.

Over the past 30 years, since the introduction of AChEIs, it has become evident that symptomatic treatments aimed at enhancing cognitive function in AD patients are largely ineffective. Furthermore, increasing neuronal activity through these treatments may accelerate neuron destruction due to overexertion, particularly with cholinergic and anti-amyloid beta (Aβ) therapies. This is especially true for carriers of the APOE-4 allele, who tend to deteriorate more rapidly and experience further cerebrovascular damage [9,17]. Therefore, the need for a paradigm shift in several ways seems clear: (i) preventive intervention in asymptomatic phases of the disease to delay neuronal death by precluding the activation of genetic/epigenetic-driven premature neurodegeneration; (ii) personalization of treatment based on the genomic profile of each patient, since the onset of the disease and the therapeutic response depend on the genomic load (Figure 2), following a golden rule in genomic medicine: the greater the number of dysfunctional genes, the earlier the onset of the disease, the faster the clinical course, and the worse the therapeutic response; and the lower the number of affected genes, the later the onset, the slower the clinical course, and the better the therapeutic response to conventional drugs [1,9,11]; (iii) development and introduction of new drugs capable of controlling and/or inhibiting the multiple pathogenic cascades that lead to the premature death of neurons; (iv) identical prophylactic approach to all concomitant diseases with a potential deleterious effect on the nervous system, capable of increasing neuronal damage (i.e., cardiovascular, cerebrovascular, metabolic, endocrine diseases, etc.).

Obtaining an effective MT product is a challenging task. Despite hundreds of attempts over the past decade (Tables S4 and S5), no MT products have successfully progressed from the preclinical to clinical phase. Most of the MT drugs developed so far are somewhat naive and redundant, based on old pharmacological concepts that have shown little efficacy: cholinesterase inhibitors, antioxidants, anti-inflammatories, anti-tau phosphorylation, and anti-Aβ deposition [223,224,225]. Since the presence of the epsilon 4 allele is an essential risk factor for AD [226,227,228], it is striking to note that hardly any attempts have been made to find MT drugs capable of modulating APOE expression.

Hybrid molecules capable of combining MT effects and selective epigenetic mechanisms to control the expression of defective genes are likely to be an option worth exploring. The classic profile of epidrugs is not very satisfactory, either due to their toxicity and narrow therapeutic window; however, both in the plant kingdom and in the marine environment, a host of new molecules are being discovered and characterized, many of them still unexplored (Table S5), which could bring a little hope to interfere with the genomic-epigenetic mechanisms that are undoubtedly primarily responsible for neurons starting to die at early ages of life until, once exhausted or dead, they cause brain functions to fail and the disease to manifest itself in old age.

NST is an interesting biotechnological paradigm of MT bioproduct, with clear anti-Aβ, anti-neuroinflammatory, antioxidant, neurotrophic, and epigenetic effects, where its powerful epigenetic effect as a DNA methylating agent, HDAC inhibitor, and sirtuin modulator justifies its regulatory capacity of AD gene expression [199,208,209]; however, the characterization of similar products and their testing in humans is complicated, saving the differences that exist in a transgenic model compared to the neuropathological reality of AD in humans.

The enormous difficulty in identifying active principles in biological products of this nature should not be ignored either. If it is found, it should not be forgotten that experience shows that the pleiotropic effects of native biological products are always superior to the effects obtained with active ingredients isolated from the molecular complex of natural origin (whether animal, marine, or plant).

Another important consideration is defining what constitutes an MT drug, including its organoleptic properties and its safety and efficacy profile. The profile of a multi-target drug with curative character for AD should meet a series of preclinical and clinical criteria: (i) An MT drug should act, at least, effectively on three or more major pathogenic pathways. (ii) Its short- and medium-term effects should be quantifiable by presymptomatic genomic and epigenetic biomarkers. (iii) In the medium and long term, its neuroprotective and anti-neurodegenerative effects should be confirmed with functional neuroimaging techniques (fMRI, PET-Scan). (iv) It should guarantee cognitive stability for decades when prescribed in asymptomatic phases and improve cognition when administered to clinically diagnosed AD patients. (v) Its administration should be easy and comfortable for the patient. (vi) Its prescription should be preceded by a pharmacogenetic screening that guarantees its safety and minimum toxicity. (vii) It should have a reasonable and affordable cost to be financed by the social security systems of any developed country to avoid an increase in social inequalities, which already exist today, in the group of elderly people where cases of dementia are concentrated.

Finally, an important element to keep in mind is that the therapeutic response to any drug, and of course any possible MT drug, depends on the individual pharmacogenetic profile of each patient (pathogenic, mechanistic, metabolic, transporters, and pleiotropic genes), such that the greater the number of defective pharmagenes, the worse the therapeutic response; consequently, for the development of anti-AD MT drugs, all clinical trials should include a pharmacogenetic protocol for the personalization of pharmacological treatment (Table S3) [9,11,229,230,231,232,233].

An additional step that will have to be taken sooner or later in AD and other neurodegenerative diseases is the molecular treatment to regulate the expression of the hundreds of defective genes that predispose to AD and whose activation/deactivation at early ages of life sets in motion the irreversible process of brain degeneration. The techniques that must be implemented include the replacement of defective genes and changing DNA sequences by gene editing techniques such as zinc finger proteins (ZFPs) and meganucleases and transcription activator-like effector nucleases (TALENs) and CRISPR/Cas9, with the ability to efficiently repair double-strand breaks (DSBs) and pathogenic SNPs at the target DNA [234,235,236,237]. The regulation of abnormalities in the expression of defective genes will have to be tackled with epigenetic drugs [189,238,239], with RNA interference (RNAi) [240], anti-sense oligonucleotides (ASOs) [241,242,243], and catalytic nucleic acids [244]. The development of MT drugs is an intermediate and complementary step to the future introduction of some of these new technologies to efficiently neutralize—not without complications and serious adversities—the multiple pathogenic cascades that lead to AD.

## Data Availability

The original contributions presented in the study are included in the article/Supplementary Material, further inquiries can be directed to the corresponding author.

## Supplementary Materials

The following supporting information can be downloaded at: https://www.mdpi.com/article/10.3390/life14121555/s1, Table S1: Tacrine-based multi-target drugs; Table S2: Donepezil-based multi-functional agents; Table S3: Pharmacological profile and pharmacogenetics of selected epigenetic drugs; Table S4: Selected multi-target drugs with potential effects in Alzheimer’s disease models; Table S5: Natural bioproducts with potential MT activity in Alzheimer’s disease models; Table S6: Abbreviations.
