# Supplementary material for: Therapeutic Options in Alzheimer’s Disease: From Classic Acetylcholinesterase Inhibitors to Multi-Target Drugs with Pleiotropic Activity
Source: Life (Basel). 2024 Nov 26;14(12):1555. doi: 10.3390/life14121555 (PMC11678002; doi:10.3390/life14121555)
Supplement: Supplementary file 1 [file life-14-01555-s001.zip › life-3304553-supplementary/Table S1.docx]

**Table 1. Tacrine-based multi-target drugs**

7-Methoxytacrine-adamantylamine heterodimers

9-Amino-1,2,3,4-tetrahydroacridine derivatives with 2-fluorobenzoic acid or 3-fluorobenzoic acid moiety

Bis-(7)-Tacrine derivatives

Capsaicin-Tacrine hybrids

Conjugates of tacrine with 1,2,4-thiadiazole derivatives

Conjugates of tacrine and salicylamide

Salicylimine derivatives

Cystamine-tacrine dimer

Cystamine-bis-(7)-tacrine hybrid

Ferulic (Lipoic) acid plus Melatonin-modified Tacrines

Methylene-linked 1,2,3,4-tetrahydrobenzo[h][1,6]naphthyridine-6-chlorotacrine hybrids

Multifunctional tacrine-donepezil hybrids

Phenylthiazole-tacrine hybrids

Quinolinetrione-tacrine hybrids

Tacrine–4-oxo-4H-chromene hybrids

Tacrine-based cyclopentapyranopyridine- and tetrahydropyranoquinoline-kojic acid derivatives

Tacrine-Adamantanes hybrids

7-MEOTA-amantadine hybrids

6-Chlorotacrine-memantine hybrid

Tacrine-Benzene/pyridine hybrid

Tacrine-Benzofuran hybrids

Tacrine–Benzotiazole derivatives

Tacrine–Bifendate hybrids

Tacrine Conjugates with 2-Arylhydrazinylidene-1,3-Diketones

Tacrine-deferiprone hybrids

Tacrine-Donepezil hybrids

Tacrine-Ferulic acid hybrids

THA–ferulic acid hybrids

THA–ferulic acid–NO-donor thihybrids

THA–ferulic acid hybrids with piperazine linker

Tacrine-flavonoid hybrids

Tacrine hybrids with HDAC Inhibitors

THA (6-Cl-THA)–HDAC inhibitors hybrids

Tacrine Hybrids with Thio Derivatives

Tacrine Hybrids with Fluorescent Probes

6-Cl-THA-5-phenylpyrano [3,2-c]quinoline hybrids

6-Cl-THA–tetrahydrobenzo[h][1,6]naphthyridine

THA–lophine hybrids

Tacrine Hybrids with Ca2+ Channel Blocker

THA–nimodipine hybrids

THA–dihydropyridine hybrid

THA-dihydropyirimidine-thione hybrids

Tacrine Hybrids with Modulators of Serotonin Receptors

THA-1-(phenylsulfonyl)-4-(piperazin-1-yl)-1H-indole hybrids

THA–5HT6-agonist hybrids

THA–Vilazodone hybrids

Tacrine Hybrids with Modulator of Muscarinic Receptors

Gallamine–THA hybrids

THA–xanomeline hybrids with amine linker

7-MEOTA–BQCA hybrids

THA–BQCA hybrids

6-Cl-THA–BQCA hybrids

THA–xanomeline hybrids

Tacrine Hybrids with Cannabinoid CB1 Receptor Antagonists

Tacrine Hybrids with Modulator of NMDA Receptors

7-MEOTA–adamantylamine hybrids

Benzohomoadamantane–6-Cl-THA hybrids

Tacrine Hybrids with Modulators of Opioid Receptors

THA–Tianeptine hybrids

Tacrine Hybrids with MAO Inhibitors

THA–selegiline hybrids

Tacrine Hybrids with Natural Products

THA–coumarin hybrids

THA–flavonoid hybrids

Huprine Y-rhein hybrids

THA-carbazoles hybrids

6-Cl-THA–Scutellarin hybrids

THA–resveratrol hybrids

THA hybrids with natural-based D-xylose, D-ribose, and and D-galactose

Tacrine-Hydroxybenzoyl-Pyridone hybrids

Tacrine-Hydroxyphenylbenzimidazole hybrids

Tacrine–Huprine hybrids

Tacrine-Ibuprofen hybrids

Tacrine-Isatin Schiff base hybrid derivatives

Tacrine–Melatonin Hybrids

Tacrine-Nimodipine hybrids

Tacrine-Resveratrol fused hybrids

Tacrine-Salicylimine derivatives

Tacrine-Scutellarin Hybrids

Tacrine-Selegiline Hybrids

Tacrine–Phenothiazine hybrids

Tacrine-1-trifluoromethoxyphenyl-3-(1-propionylpiperidin-4-yl) urea (TPPU) hybrids

6-Cl-THA–TPPU

Huprine–TPPU hybrids

Tacrine-(β-Carboline) hybrids

Tacripyrines

THA–antioxidant CR-6 hybrids

THA–(b-carbolines (pyrido [3,4-b]indoles) hybrids

THA–Caffeic acid hybrids

THA–Ebselen hybrids

THA–huperzine A hybrids

Huprine A

THA hybrids with N,N-dimethylated flavonoids

THA hybrids with NO-donating moieties

THA–(hydroxybenzoyl-pyridone) hybrids

THA–hydroxyquinoline hybrids

THA-NSAID hybrids

THA–flurbiprofen hybrids

THA–flurbiprofen–NO–donating hybrids

THA–indometacine hybrids

ROS–responsive ibuprofen–THA hybrids

THA–phenolic acid dihybrids

THA–phenolic acid–ligustrazine trihybrids

THA–Silibinin hybrids

THA–Triazole–chalkone conjugates

THA–Trolox hybrids

THA derivatives (miscelaneous)

Huprine Y derivatives

Huprine–based hybrids with 2-(2-oxopyrrolidin-1-yl)butyramide moiety of levetiracetam

THA-tryptophan hybrids

THA-indole hybrids

THA-based hybrids with anacardic acid, Cardanol and Cardols

THA-trimethoxybenzene hybrids

Photoswitchable hybrids

Quinone–THA hybrids

THA-propargylamine hybrids

7-MEOTA-p-anisidine hybrids

THA-1,2,3-triazole hybrids

Schiff base hybrids

THA-pyrimidone hybrids

THA–carbamate hybrids

THA hybrids with phosphorus moieties
