# Supplementary material for: Therapeutic Options in Alzheimer’s Disease: From Classic Acetylcholinesterase Inhibitors to Multi-Target Drugs with Pleiotropic Activity
Source: Life (Basel). 2024 Nov 26;14(12):1555. doi: 10.3390/life14121555 (PMC11678002; doi:10.3390/life14121555)
Supplement: Supplementary file 1 [file life-14-01555-s001.zip › life-3304553-supplementary/Table S2.docx]

**Table 2. Donepezil-based multi-functional agents**

1-Aryldonepezil analogs

Aromatic amino hybrids

Azido 1,2,3-triazoles-benzylpiperidine analog

Cinnamoyl-N-Acylhydrazone-Donepezil hybrids

Dimethylbenzimidazolinone–benzylpiperidine hybrid

Donecopride-RS67333-Donepezil hybrid

Donepezil-Arylsulfonamide Hybrids

Donepezil-based benzylamine derivatives.

Donepezil + Chromone + Melatonin hybrids

Donepezil–ferulic acid hybrids

Donepezil–flavonoid hybrids

Donepezil-8-hydroxyquinoline derivative

Donepezil-Hydroxytyrosol derivatives

Donepezil–indole hybrids

Donepezil-Melatonin derivatives

Donepezil-PF9601N hybrids

Donepezil-Propargylamine-8-hydroxyquinoline hybrids

E2020-NOH

Feruloyl-donepezil hybrids

Fused Donepezil-Curcumin derivatives

Hydroxy Benzimidazole-Donepezil hybrids

Isoxazole/oxadiazole–benzylpiperidine hybrids

Lipoic acid-based benzylpiperidine hybrid

N-Nenzyl-piperidinyl-aryl-acylhydrazone derivatives-donepezil hybrids

NSAID-Donepezil conjugates

Phenothiazine/Donepezil-like Hybrids

Phenylpyridazine-3-carboxamide-benzylpiperidine hybrid

Phthalazin-1(2H)-one-donepezil hybrid.

Phthalimide-dithiocarbamate hybrids

Pyrazole-benzylpiperidine derivative

Pyridine-donepezil hybrids

Pyrrolizine–benzylpiperidine hybrid.

Quinolone–benzylpiperidine derivative

Racemic Trans Propargylamino-Donepezil

Tenuazonic-donepezil hybrids

Thiazole-benzylpiperidine scaffolds

Vilazodone-Donepezil chimeras
