# Supplementary material for: Therapeutic Options in Alzheimer’s Disease: From Classic Acetylcholinesterase Inhibitors to Multi-Target Drugs with Pleiotropic Activity
Source: Life (Basel). 2024 Nov 26;14(12):1555. doi: 10.3390/life14121555 (PMC11678002; doi:10.3390/life14121555)
Supplement: Supplementary file 1 [file life-14-01555-s001.zip › life-3304553-supplementary/Table S3.docx]

**Table 3. Pharmacological profile and pharmacogenetics of selected epigenetic drugs**

| **Properties** | **Pharmacogenetics** |
| --- | --- |
| **Name:** **5-Azacytidine**, Azacitidine, Azacytidine, Ladakamycin, Vidaza, Mylosar, Azacitidinum, 5-AZAC  **IUPAC Name:** 4-Amino-1-[(2R,3R,4S,5R)-3,4-dihydroxy-5-(hydroxymethyl)oxolan-2-yl]-1,3,5-triazin-2-one  **Molecular Formula:** C_8_H_12_N_4_O_5_  **Molecular Weight:** 244.20 g/mol  **Category:** Pyrimidine nucleoside cytidine analog  **Mechanism:** DNA methyltransferase inhibitor, Telomerase inhibitor  **-Target:** [DNA (cytosine-5)-methyltransferase 1](http://www.drugbank.ca/molecules/1064?as=target) (DNMT1)  **-Interactions:** [Cytidine deaminase](http://www.drugbank.ca/molecules/4211?as=enzyme)  **Effect:** Antineoplastic, Antimetabolite. Methylates CpG residues. Methylates hemimethylated DNA. Mediates transcriptional repression by direct binding to HDAC2 | **Pathogenic genes:** *ALDH3A1, CDKN2A, MGMT, PLA2R1, RRM1, TNFRSF1B*  **Mechanistic genes:** *ALDH1A1, DAPK1, DNMT1, DPYD, CDKN2A, MGMT, PLCB1*  **Metabolic genes:**  **Substrate:** *CDA, DCK, SLC28A1, SLC29A1, RRM1, RRM2, UCK1, UCK2*  **Inhibitor:** *CYP1A2 (weak), CYP2E1 (weak), DNMT1*  **Inducer:** *SULT1C2*  **Transporter genes:** *SLC5A5,* *SLC28A1,* [*SLC29A1*](http://www.ncbi.nlm.nih.gov/gene/2030)  **Pleiotropic genes:** *BLK* |
| **Name: Curcumin**, Diferuloylmethane, Natural yellow 3, Turmeric yellow, Turmeric, Kacha haldi, Gelbwurz, Curcuma, Haldar, Souchet  **IUPAC Name:** (1E,6E)-1,7-bis(4-hydroxy-3-methoxyphenyl)hepta-1,6-diene-3,5-dione  **Molecular Formula:** C_21_H_20_O_6_  **Molecular Weight:** 368.38 g/mol  **Category:** Natural product (*Curcuma longa)*  **Mechanism:** Histone acetyltransferase (HAT) inhibitor  **Effect:** Non-steroidal anti-inflammatory agent; Antineoplastic; Antioxidant; Cognitive enhancer; Coloring agent; Enzyme inhibitor | **Pathogenic genes:** *BACE1, CCND1, CDH1, GSK3B, IL1A, IL6, JUN, MSR1, PSEN1, PTGS2, SNCA, SREBF1, TNF*  **Mechanistic genes:** *AKT1,* *PRKAs, BACE1, CCND1, CDH1, CDKs, CRM1, CTNNB1, EGF, GSK3B, HDACs, HIF1A, IL1A, IL6, JUN, MMPs, MSR1, NFKB1, NOS2, PDGFRs, PSEN1, PTGS2, SNCA, SOCS1, SOCS3, SREBF1, STAT3, TNF, VEGFA*  **Metabolic genes:**  **Inhibitor:** *CYP2C8, CYP2C9, EP300*  **Inducer:** *CYP2C8, CYP2C9, CYP2D6, CYP3A4*  **Transporter genes:** *ABCA1, SNCA*  **Pleiotropic genes:** *CTNNB1, MSR1* |
| **Name:** [**Decitabine**](http://www.ncbi.nlm.nih.gov/sites/entrez?cmd=search&db=pcsubstance&term=%22Decitabine%22%5bCompleteSynonym%5d%20451668%5bstandardizedcid%5d), [5-Aza-2'-deoxycytidine](http://www.ncbi.nlm.nih.gov/sites/entrez?cmd=search&db=pcsubstance&term=%225%2dAza%2d2%26%2339%2b%2ddeoxycytidine%22%5bCompleteSynonym%5d%20451668%5bstandardizedcid%5d), [Dacogen](http://www.ncbi.nlm.nih.gov/sites/entrez?cmd=search&db=pcsubstance&term=%22Dacogen%22%5bCompleteSynonym%5d%20451668%5bstandardizedcid%5d), [Dezocitidine](http://www.ncbi.nlm.nih.gov/sites/entrez?cmd=search&db=pcsubstance&term=%22Dezocitidine%22%5bCompleteSynonym%5d%20451668%5bstandardizedcid%5d), [2'-Deoxy-5-azacytidine](http://www.ncbi.nlm.nih.gov/sites/entrez?cmd=search&db=pcsubstance&term=%222%26%2339%2b%2dDeoxy%2d5%2dazacytidine%22%5bCompleteSynonym%5d%20451668%5bstandardizedcid%5d)  **IUPAC Name:** 4-Amino-1-[(2R,4S,5R)-4-hydroxy-5-(hydroxymethyl)oxolan-2-yl]-1,3, 5-triazin-2-one  **Molecular Formula:** C_8_H_12_N_4_O_4_  **Molecular Weight:** 228.21 g/mol  **Category:** Nucleoside  **Mechanism:** DNA methyltransferase inhibitor  -**Target:** [DNA (cytosine-5)-methyltransferase 1](http://www.drugbank.ca/molecules/1064?as=target) (DNMT1)  -**Interactions:** [Deoxycytidine kinase](http://www.drugbank.ca/molecules/4773?as=enzyme)  **Effect:** Antineoplastic, Antimetabolite, Enzyme inhibitor, Teratogen | **Pathogenic genes:** *BRCA1, CDKN2B, DNMT3A, EGFR, FOS, MGMT, MLH1, MMP9, MYC, NOS3, NQO1, TP53, VHL*  **Mechanistic genes:** *APAF1, BRCA1, CDKN2B, EGFR, ICAM1, MAGED1, MGMT, MLH1, MMP2, MMP9, MYC, NOS3, TIMP3, TP53, VHL, ZNF350.*  **Metabolic genes:**  **Substrate*:*** *DCK, DNMT1, CDA, SLC29A1*  **Inhibitor*:*** *DNMT1, DNMT3B*  **Inducer:** *DPYD*  **Transporter genes:** *ABCs, SLC15s, SLC22s, SLC28A1, SLC29As*  **Pleiotropic genes:** *HBG1, NQO1, NTRK2, MMP2, MSH2* |
| **Name: Epigallocatechin 3-gallate, EGCG**, (-)-epigallocatechin gallate, tea catechin, teavigo, catechin deriv., 989-51-5 **IUPAC name:** [(2R,3R)-5,7-dihydroxy-2-(3,4,5-trihydroxyphenyl)-3,4-dihydro-2H-chromen-3-yl] 3,4,5-trihydroxybenzoate  **Molecular formula:** C_22_H_18_O_11_  **Molecular Weight:** 458.37 g/mol  **Category:** DNMT inhibitors  **Targets:** DNMT1 | **Pathogenic genes:**  *APP, BACE1, CDX2, EGFR, FAS, PIK3CA, ROS1*  **Mechanistic genes:**  *APP, BACE1, BMP2, CDX2, CHRNA7, ECEs, EGFR, IRS1, PIK3CA, ROS1*  **Metabolic genes:**  **Inhibitor:** *SOD*  **Transporter genes:**  *CD36, SLC5A1, SLC27A4, SLCO1B1, SLCO1B3*  **Pleiotropic genes:**  *ACACA, CHRNA7, SCD* |
| **Name:** **Entinostat**, ms-275, 209783-80-2, SNDX-275, MS 275, MS-27-275, SNDX 275, Histone Deacetylase Inhibitor I, S1053_Selleck, MS 27-275  **IUPAC Name:** Pyridin-3-ylmethyl N-[[4-[(2-aminophenyl)carbamoyl]phenyl]methyl]carbamate  **Molecular Formula:** C_21_H_20_N_4_O_3_  **Molecular Weight:** 376.41 g/mol  **Category:** Benzamide  **Mechanism:** Class I HDAC inhibitor (HDAC1, 2, 3)  **Effect:** Antineoplastic agent; Histone deacetylase inhibitor; Memory enhancer | **Pathogenic genes:** *CDH1*  **Mechanistic genes:** *CDH1, HDAC1, HDAC2, HDAC3, KLRK1*  **Metabolic genes:**  **Inhibitor:** *HDAC1, HDAC2, HDAC3*  **Inducer:** *CYP19A1* |
| **Name:** **Mocetinostat**, MGCD0103, 726169-73-9, MGCD-0103, MGCD 0103, N-(2-Aminophenyl)-4-([[4-(pyridin-3-yl)pyrimidin-2 yl]amino]methyl)benzamide  **IUPAC Name:** N-(2-Aminophenyl)-4-[[(4-pyridin-3-ylpyrimidin-2-yl)amino]methyl] benzamide  **Molecular Formula:** C_23_H_20_N_6_O  **Molecular Weight:** 396.44 g/mol  **Category:** Benzamide  **Mechanism:** Class I HDAC inhibitor (HDAC1, 2, 3); Class IV HDAC inhibitor (HDAC11)  **Effect:** Antineoplastic agent; Histone deacetylase inhibitor | **Pathogenic genes:** *CDKN1A*, *CDKN2B, TNF*  **Mechanistic genes:** *CDKN1A, CDKN2B, HDAC1, HDAC2, HDAC3, HDAC11, NFKB2, TNF*  **Metabolic genes:**  **Inhibitor:** *HDAC1, HDAC2, HDAC3,HDAC11* |
| **Name:** **Nicotinamide**, niacinamide, vitamin PP, aminicotin, nicotinic acid amide, amixicotyn, 3-pyridinecarboxamide, papulex, nicotylamide  **IUPAC name:** pyridine-3-carboxamide  **Molecular formula:** C_6_H_6_N_2_O  **Molecular Weight:** 122.12 g/mol  **Category:** SIRT inhibitors  **Targets:** class III HDAC (SIRT1-7) | **Pathogenic genes:**  *IL6, IL8, PTGS2, TNF*  **Mechanistic genes:**  *ARTs, CAT, CLOCK, FOXO3, GPXs, IL6, IL8, PARP1, PTGS2, SIRT1, SOD1, TNF*  **Metabolic genes:**  **Inhibitor:** *CYP2D6, CYP3A4, CYP2E1, SIRT1-7*  **Pleiotropic genes:**  *CAT, PARP1* |
| **Name: Panobinostat**, LBH-589, 404950-80-7, LBH589, Faridak, NVP-LBH589, LBH 589, S1030_Selleck, AC1OCFY8, Panobinostat (LBH589)  **IUPAC Name:** (E)-N-hydroxy-3-[4-[[2-(2-methyl-1H-indol-3-yl)ethylamino]methyl]phenyl] prop-2-enamide  **Molecular Formula:** C_21_H_23_N_3_O_2_  **Molecular Weight:** 349.43 g/mol  **Category:** Hydroxamic acid  **Mechanism:** Class I HDAC inhibitor (HDAC1, 2, 3, 8); Class IIa HDAC inhibitor (HDAC4, 5, 7, 9); Class IIb HDAC inhibitor (HDAC6, 10); Class IV HDAC inhibitor (HDAC11); Pan-histone deacetylase inhibitor  **Effect:** Antineoplastic agent; Histone deacetylase inhibitor | **Pathogenic genes:** *CDKN1A*, *EGFR, IL6, RASSF1*  **Mechanistic genes:** *AKT1, CDKN1A, DAPK1, DNMT1, EGFR, HDACs, HIST3H3, HIST4H4, HSP90As, IL6, IL10, IL12, IL23A, NFKB2, RASSF1,* *TLR3*  **Metabolic genes:**  **Substrate:** *CYP2C19, CYP2D6, CYP3A4*  **Inhibitor:** *AKT1, CYP19A1 (strong), HDACs*  **Pleiotropic genes:** *IL10* |
| **Name:** **Pivanex**, AN-9, Pivalyloxymethyl butyrate, AN 9, 122110-53-6, BRN 4861411, ((2,2 Dimethylpropanoyl)oxy)methyl butanoate  **IUPAC Name:** Butanoyloxymethyl 2,2-dimethylpropanoate  **Molecular Formula:** C_10_H_18_O_4_  **Molecular Weight:** 202.25 g/mol  **Category:** Short-chain fatty acid  **Mechanism:** Class I HDAC inhibitor (HDAC1, 2, 3, 8)  **Effect:** Antineoplastic agent; Histone deacetylase inhibitor | **Pathogenic genes:** *BCL2, TP53*  **Mechanistic genes:** *BAX, BCL2, BCR-ABL, HDACs, TP53*  **Metabolic genes:**  **Inhibitor:** *ABCB1, HDACs*  **Transporter genes:** *ABCB1* |
| **Name:** **Quercetin**; Sophoretin; Quercetol; Meletin; Xanthaurine; Quercitin; 3,3',4',5,7-Pentahydroxyflavone  **IUPAC name:** 2-(3,4-dihydroxyphenyl)-3,5,7-trihydroxychromen-4-one  **Molecular formula:** C_15_H_10_O_7_  **Molecular Weight:** 302.24 g/mol  **Category:** DNMT inhibitors  **Targets:** DNMT1 | **Pathogenic genes:**  *IL1R, NFkB, Ccl8, IKK, STAT3, CD4, CDK2, IL2*  **Mechanistic genes:**  *MTND4, CDKN2A, PRDX4, DIO2, HSD17B1, MSH2, GSS, COMT, FOS, CRP, NR1I3, PON1*  **Metabolic genes:**  **Substrate:** *UGT1A1, UGT1A3, GSTT1, CYP2J2, GSTK1, CYP2C8, CYP1A1, CYP1A2, CYP1B1, GSTA1, CYP19A1*  **Inhibitor:** *SULT1E1*  **Transporter genes:**  *ABCB1, ABCG2* |
| **Name: Resveratrol**, trans-resveratrol, 501-36-0, 3,4',5-Trihydroxystilbene, 3,4',5-Stilbenetriol, 3,5,4'-Trihydroxystilbene, Resvida, (E)-resveratrol  **IUPAC Name:** 5-[(E)-2-(4-Hydroxyphenyl)ethenyl]benzene-1,3-diol  **Molecular Formula:** C_14_H_12_O_3_  **Molecular Weight:** 228.24 g/mol  **Category:** Natural polyphenol  **Mechanism:** SIRT1 inducer/activator  **Effect:** Non-steroidal antiinflammatory agent; Anticarcinogenic; Antimutagenic; Antineoplastic; Antioxidant; Platelet aggregation inhibitor; Enzyme inhibitor; Lifespan extension; Memory improvement; Aβ decrease; Reduction of plaque formation | **Pathogenic genes:** *BCL2, CAV1, ESR1, ESR2, GRIN2B, NOS3, PTGS2, TNFRSF10A, TNFRSF10B*  **Mechanistic genes:** *APP, ATF3, BAX, BAK1, BBC3, BCL2, BCL2L1, BCL2L11, BIRC5, CASP3, CAV1, CFTR, ESR1, ESR2, GRIN1, GRIN2B, HTR3A, NFKB1, NOS3, PMAIP1, PTGS1, PTGS2, SIRT1, SIRT3, SIRT5, SRC, TNFRSF10A, TNFRSF10B, TRPs*  **Metabolic genes:**  **Substrate:** *CYP1A1, CYP1A2, CYP1B1, CYP2E1, GSTP1, PTGS1, PTGS2*  **Inhibitor:** *CYP1A1, CYP1B1, CYP2C9, CYP2D6, CYP3A4, NQO2*  **Inducer:** *CYP1A2, SIRT1*  **Transporter genes:** *ABCC1, ABCC2, ABCC3, ABCC4, ABCC8, ABCG1, ABCG2, CFTR, TRPs* |
| **Name:** **Romidepsin**, Depsipeptide, Chromadax, Istodax, Antibiotic FR 901228, FK228, FR 901228, FK-228, NSC 630176, NSC-630176  **IUPAC Name:** (1S,4S,7Z,10S,16E,21R)-7-ethylidene-4,21-di(propan-2-yl)-2-oxa-12, 13-dithia-5,8,20,23-tetrazabicyclo[8.7.6]tricos-16-ene-3,6,9,19, 22-pentone  **Molecular Formula:** C_24_H_36_N_4_O_6_S_2_  **Molecular Weight:** 540.70 g/mol  **Category:** Cyclic peptide  **Mechanism:** Class I HDAC inhibitor (HDAC1, 2, 3, 8); Class IIa HDAC inhibitor (HDAC4,5,7,9); Class IIb HDAC inhibitor (HDAC6, 10); Class IV HDAC inhibitor (HDAC11)  **Effect:** Antibiotic; Antineoplastic agent; Histone deacetylase inhibitor | **Pathogenic genes:** *BCL2, CCDN1*, *CDKN1A*, *MYC, NF2, RB1, ROS1, TNFSF10, VHL*  **Mechanistic genes:** *BCL2, CCDN1, CDKN1A, FLT1, HDAC1, HDAC2, HDAC3, HDAC4, HSP90As, KDR, MYC, NF2, TNFSF10, VEGFs, VHL*  **Metabolic genes:**  **Substrate:** *ABCB1, ABCG2, CYP1A1 (minor), CYP2B6 (minor), CYP2C19 (minor), CYP3A4 (major), CYP3A5 (minor), NR1I3, SLCO1B3*  **Inhibitor:** *ABCB1, HDACs*  **Inducer:** *ABCG2*  **Transporter genes:** *ABCB1, ABCC1, ABCG2, SLCO1B3*  **Pleiotropic genes:** *CDH1, CDKN1A* |
| **Name: S-Adenosylmethionine**, Ademetionine, AdoMet, Donamet, S-adenosyl-L-methionine, SAMe, Methioninyladenylate, SAM-e, adenosylmethionine  **IUPAC Name:** (2S)-2-Amino-4-[[(2S,3S,4R,5R)-5-(6-aminopurin-9-yl)-3,4-dihydroxyoxolan-2-yl]methyl-methylsulfonio]butanoate  **Molecular Formula:** C_15_H_22_N_6_O_5_S  **Molecular Weight:** 398.44 g/mol  **Category:** Methyl radical donor  **Mechanism:** Histone methyltransferase inhibitor  **Effect:** Antineoplastic; Antiinflammatory; Memory enhancer;  PSEN1 repressor | **Pathogenic genes:** *AKT1, ERK, GNMT, MAT1A, PSEN1*  **Mechanistic genes:** *AMD1, CAT, CBS, GCLC, GNMT, GSS, NOS2, ROS1, STAT1, TNF*  **Metabolic genes:**  **Substrate:** *COMT, GNMT, TPMT, SRM*  **Inhibitor:** *ABCB1, CYP2E1, NOS2*  **Transporter genes:** *SLC25A26*  **Pleiotropic genes:** *CAT, TNF* |
| **Name: Sodium phenylbutyrate**, [Buphenyl,](http://dailymed.nlm.nih.gov/dailymed/lookup.cfm?setid=6a05ace2-3d5e-48de-b158-870d404cc061) 4-Phenylbutiric acid, 4-Phenylbutanoic acid, Benzenebutanoic acid, Benzenebutyric acid, Butyric acid, 4-phenyl-, 1821-12-1, gamma-Phenylbutyric acid,  **IUPAC Name:** 4-Phenylbutanoic acid  **Molecular Formula:** C_10_H_12_O_2_  **Molecular Weight:** 164.20 g/mol  **Category:** Short-chain fatty acid  **Mechanism:** Class I HDAC inhibitor (HDAC1, 2, 3, 8); Class IIa inhibitor (HDAC4,5,7,9); Class IIb inhibitor (HDAC6,10)  **Effect:** Antineoplastic agent; Histone deacetylase inhibitor; Memory improvement; pTau decrease via GSK3β inactivation; C99 and Aβ decrease; Amyloid burden reduction | **Pathogenic genes:** *ARG1, ASS1, BCL2, CPS1, NAGS, OTC*  **Mechanistic genes:** *BCL2, BDNF, EDN1, HDACs, HSPA8, ICAM1, NFKB2, NT3, VCAM1*  **Metabolic genes:**  **Inhibitor:** *HDACs*  **Inducer:** *ARG1, CFTR, CYP2B6, NFKB2*  **Transporter genes:** *CFTR*  **Pleiotropic genes:** *ASL, BDNF, VCAM1* |
| **Name: Suramin**, Naphuride, Germanin, Naganol, Belganyl, Fourneau, Farma, Antrypol, Suramine, Naganin  **IUPAC Name:** 8-[[4-methyl-3-[[3-[[3-[[2-methyl-5-[(4,6, 8-trisulfonaphthalen-1-yl)carbamoyl]phenyl]carbamoyl]phenyl] carbamoylamino]benzoyl]amino]benzoyl]amino]naphthalene-1,3,5-trisulfonic acid  **Molecular Formula:** C_51_H_40_N_6_O_23_S_6_  **Molecular Weight:** 1297.28 g/mol  **Category:** Polyanionic compound  **Mechanism:** Class III HDAC/Sirtuin inhibitor (SIRT1-3)  **Effect:** Antineoplastic Agent; Trypanocidal Agent; Antiparasitic; Antinematodal (African trypanosomiasis, Onchocerca); Sirtuin inhibitor | **Mechanistic genes:** *FSHR, IL10, P2RY2, PDGFRB, RYR1, SIRT1,SIRT2, SIRT3, SIRT5*  **Metabolic genes:**  **Inhibitor:** *SIRT1, SIRT2, SIRT3* |
| **Name: Trichostatin A**, 58880-19-6, TSA, Trichostatin A (TSA), CHEBI:46024, TSA; [2,4-Heptadienamide, 7-(4-(dimethylamino)phenyl)-N-hydroxy-4,6-dimethyl-7-oxo-](http://www.ncbi.nlm.nih.gov/sites/entrez?cmd=search&db=pcsubstance&term=%222%2c4%2dHeptadienamide%2c%207%2d(4%2d(dimethylamino)phenyl)%2dN%2dhydroxy%2d4%2c6%2ddimethyl%2d7%2doxo%2d%22%5bCompleteSynonym%5d%20444732%5bstandardizedcid%5d) [7-(4-(Dimethylamino)phenyl)-N-hydroxy-4,6-dimethyl-7-oxo-2,4-heptadienamide](http://www.ncbi.nlm.nih.gov/sites/entrez?cmd=search&db=pcsubstance&term=%227%2d(4%2d(Dimethylamino)phenyl)%2dN%2dhydroxy%2d4%2c6%2ddimethyl%2d7%2doxo%2d2%2c4%2dheptadienamide%22%5bCompleteSynonym%5d%20444732%5bstandardizedcid%5d); [[R-(E,E)]-7-[4-(Dimethylamino)phenyl]-N-hydroxy-4,6-dimethyl-7-oxo-2,4-heptadienamide](http://www.ncbi.nlm.nih.gov/sites/entrez?cmd=search&db=pcsubstance&term=%22%5bR%2d(E%2cE)%5d%2d7%2d%5b4%2d(Dimethylamino)phenyl%5d%2dN%2dhydroxy%2d4%2c6%2ddimethyl%2d7%2doxo%2d2%2c4%2dheptadienamide%22%5bCompleteSynonym%5d%20444732%5bstandardizedcid%5d)  **IUPAC Name:** (2E,4E,6R)-7-[4-(dimethylamino)phenyl]-N-hydroxy-4,6-dimethyl-7-oxohepta-2,4-dienamide  **Molecular Formula:** C_17_H_22_N_2_O_3_  **Molecular Weight:** 302.37 g/mol  **Category:** Hydroxamic acid  **Mechanism:** Class I HDAC inhibitor (HDAC1, 2, 3); Class IIa HDAC inhibitor (HDAC4, 7, 9); Class IIb inhibitor (HDAC6)  **Effect:** Antifungal agent; Antibacterial agent; Histone deacetylase inhibitor; Protein synthesis inhibitor; Antineoplastic; Memory improvement; Rescue of CA3-CA1 LTP in APP/PS1 transgenic models | **Pathogenic genes:** *BCL2*  **Mechanistic genes:** *BCL2, HDACs, IL8, IL12A,IL12B, NFKB2, RARB*  **Metabolic genes:**  **Substrate:** *CYP3A4 (mayor)*  **Inhibitor:** *HDACs*  **Inducer:** *CYP1A1, CYP1B1, CYP2B6, CYP2E1, CYP7A1, SLC19A3*  **Transporter genes:** [*SLC19A3*](http://www.ncbi.nlm.nih.gov/gene/80704) |
| **Name: Valproic Acid**, 2-Propylpentanoic acid, Depakene, Depakine, Ergenyl, Dipropylacetic acid, Mylproin, Convulex, Myproic Acid  **IUPAC Name:** 2-Propylpentanoic acid  **Molecular Formula:** C_8_H_16_O_2_  **Molecular Weight:** 144.21 g/mol  **Category:** Short-chain fatty acid  **Mechanism:** Class I HDAC inhibitor (HDAC1, 2, 3, 8)  **Effect:** Anticonvulsant; Mood stabilizer; Antimanic agent; Enzyme inhibitor; Histone deacetylase inhibitor; GABA modulator; Memory improvement; Aβ and pTau decrease; CDK5 inactivation | **Pathogenic genes:** *CREB1, IL6, LEP, SCN2A, TGFB1, TNF, TRNK*  **Mechanistic genes:** *ABAT, CDK5, GSK3B, HDAC1, HDAC2, HDAC3, HDAC8, HDAC9, LEP, LEPR, SCNs, SMN2*  **Metabolic genes:**  **Substrate:** *ABCB1, CYP1A1 (minor), CYP2A6 (major), CYP2B6 (minor), CYP2C9 (major), CYP2C19 (minor), CYP2E1 (minor), CYP3A4 (minor), CYP4B1 (major), CYP4F2 (minor), UGT1A4, UGT1A6, UGT1A8, UGT1A9, UGT1A10, UGT2B7*  **Inhibitor:** *ABCB1, ACADSB, AKR1A1, CYP2A6 (moderate), CYP2C9 (strong), CYP2C19 (moderate), CYP2D6 (weak), CYP3A4 (moderate), HDAC1, HDAC2, HDAC3, HDAC8, HDAC9, UGT1A9, UGT2B1, UGT2B7*  **Inducer:** *ABCB1, AKR1C4, CASR, CYP2A6, CYP2B6, CYP3A4, CYP7A1, MAOA, NR1I2, SLC5A5, SLC6A2, SLC12A3, SLC22A16*  **Transporter genes:** *ABCB1, ABCC2, ABCG1, ABCG2, SCNs, SLC5A5, SLC6A2, SLC12A3, SLC22A16*  **Pleiotropic genes:** *ABL2, AGPAT2, ASL, ASS1, CDK4, CHRNA1, COL1A1, CPS1, CPT1A, DRD4, FMR1, FOS, HBB, HFE, HLA-A, HLA-B, ICAM1, IFNG, IL6, IL10, LEPR, NAGS, NR3C1, OTC, PTGES, STAT3, TGFB1, TNF, TP53.* |
| **Name:** **Vorinostat**, Suberoylanilide hydroxamic acid (SAHA), Zolinza, Suberanilohydroxamic acid, 149647-78-9, N-hydroxy-N'-phenyloctanediamide, SAHA cpd  **IUPAC Name:** N'-Hydroxy-N-phenyloctanediamide  **Molecular Formula:** C_14_H_20_N_2_O_3_  **Molecular Weight:** 264.32 g/mol  **Category:** Hydroxamic acid  **Mechanism:** Class I HDAC inhibitor (HDAC1, 2, 3, 8)  Class IIb inhibitor (HDAC6)  **Effect:** Antineoplastic, Memory improvement | **Pathogenic genes:** BIRC3, CCND1, *CDKN1A*, CFLAR, CYP19A1, ERBB2, ERBB3, EGFR, RB1, TP53, TNF  **Mechanistic genes:** *CDK*N1A, EGFR, ERBB2, ERBB3, STATs, TYMS, VEGFs  **Metabolic genes:**  **Substrate:** *CYP2A6 (minor), CYP2C9 (minor), CYP2C19 (major), CYP2D6 (minor), CYP3A4 (major)*  **Inhibitor:** *HDAC1, HDAC2, HDAC3, HDAC6*  **Inducer:** *CYP1A1, CYP1A2, CYP1B1*  **Pleiotropic genes:** *ALPs, TNF, TYMS* |

For Abbreviations see Table S3
