# Supplementary material for: Therapeutic Options in Alzheimer’s Disease: From Classic Acetylcholinesterase Inhibitors to Multi-Target Drugs with Pleiotropic Activity
Source: Life (Basel). 2024 Nov 26;14(12):1555. doi: 10.3390/life14121555 (PMC11678002; doi:10.3390/life14121555)
Supplement: Supplementary file 1 [file life-14-01555-s001.zip › life-3304553-supplementary/Table S4.docx]

**Table S4. Selected multi-target drugs with potential effects in Alzheimer’s disease models**

(-)-Meptazinol carbamates

1,5-Diarylimidazoles

1-Methyl-4-propan-2-ylbenzene

1-Phenyl-3-hydroxy-4-pyridinone derivatives

2,2'-Bipyridyl derivatives

2,4-Dihydropyrano[2,3-c]pyrazoles

2-[4-(4-substitutedpiperazin-1-yl)phenyl]benzimidazole derivatives

2-Arylbenzofuran derivatives

2-Arylethenylquinoline derivatives

2-Arylethenyl-N-methylquinolinium derivatives

2'-Hydroxychalcones

2-Propargylamino-naphthoquinone derivatives

2-Substituted benzo[d]oxazol-5-amine derivatives

3-Arylbenzofuranone derivatives

4-Phenylbutyrate

5-Methoxyisatin 3-(4-isopropylphenyl)hydrazone

6-Methyluracil derivatives

8-Hydroxyquinoline derivatives

20(R)-Panaxadiol derivatives

Acridine

Alkyl-substituted 4-methoxy benzaldehyde thiosemicarbazones

Amiridine hybrids

Anti-Neuroinflammatory agents

Anti-inflammatory kinase signaling

NE3107

MW150

Neflamapimod/VX-745

Baricitinib)

Inhibitors of cytokines or eicosanoids

XPro1595/Pegipanermin

Canakinumab

Lenalidomide

Emtricitabine

Montelukast

Salsalate

ALZT-OP1

Modulators of microglia and astrocytes activation

AL002

TB006

Edicotinib

Sargramostim

Pepinemab

Daratumumab

Immunomodulatory agents

VT301/GB301

Cathepsin B inhibitors

Phosphatase 2 inhibitors

Monoglycerol lipase inhibitors

AOP19078710

AP2238 derivatives

AP2469

Apigenin-rivastigmine hybrids

ARN14140 (Memantine/Galantamine-Based Multi-Target Compound)

ASS234

Aurone Mannich base derivatives

AVN-101

Bakuchiol derivatives

BAS00314308

BDD26909696

Benzazepine derivatives, with the core structure of 3-methyltetrahydro-3H-benzazepin-2-one

Benzimidazole-based Schiff Base hybrid scaffolds

Benzo- and Thienobenzo-diazepines

Benzofurans

Benzyl pyridinium-2,4-dioxochroman derivatives

Biomimetic Dendrimer-peptide conjugates

Bulbocodin D

C1213

Caffeic acid derivatives

Carvelodiol derivatives

Cerebrolysin

Chiral pyrrolidines

Chromen-4-one based compounds

Chromone derivatives

α-Aminophosphonate-functionalized Chromone

Chromone-Donepezil hybrids

Chromone 2-phenylcarboximide derivatives

Chromone-3-phenylcarboximide derivatives

Chrysin derivatives

Cinnamic acid derivatives

Cinnamamide-dibenzylamine hybrids

Cinnamic acid-tryptamine hybrids

Cinnamic-N-benzylpiperidine hybrids

Cinnamic-N,N-dibenzyl(N-methyl)amine hybrids

(-)-Clausenamide

Clioquinol-1-benzyl-1,2,3,6-tetrahydropyridine hybrids

CNI-1493

Curcumin

Demethoxycurcumin

Disdemethoxycurcumin

Curcumin-Tacrine hybrids

Curcumin-Donepezil hybrids

Curcumin-Rivastigmine hybrids

Coumarin hybrids

Coumarin-Chromone-3-phenyl carboxamide

Coumarin-Pargyline hybrids

Coumarin-Pyridine Hybrids

3-Phenylcoumarin derivatives

Cyanobiphenyls

Cyclopentaquinoline hybrids

Dauricine-Graphene oxide.

Deoxyvasicinone-indole

Dihydropyrimidinone (DHPM)-derived selenoesters

Dimethyl fumarate plus Tranilast modified Dithiocarbate

Donecopride derivates

Donepezil based multi-functional agents

1-Aryldonepezil analogs

Aromatic amino hybrids

Azido 1,2,3-triazoles-benzylpiperidine analog

Cinnamoyl-N-Acylhydrazone-Donepezil hybrids

Dimethylbenzimidazolinone–benzylpiperidine hybrid

Donecopride-RS67333-Donepezil hybrid

Donepezil-Arylsulfonamide Hybrids

Donepezil-based benzylamine derivatives.

Donepezil + Chromone + Melatonin hybrids

Donepezil–ferulic acid hybrids

Donepezil–flavonoid hybrids

Donepezil-8-hydroxyquinoline derivative

Donepezil-Hydroxytyrosol derivatives

Donepezil–indole hybrids

Donepezil-Melatonin derivatives

Donepezil-PF9601N hybrids

Donepezil-Propargylamine-8-hydroxyquinoline hybrids

E2020-NOH

Feruloyl-donepezil hybrids

Fused Donepezil-Curcumin derivatives

Hydroxy Benzimidazole-Donepezil hybrids

Isoxazole/oxadiazole–benzylpiperidine hybrids

Lipoic acid-based benzylpiperidine hybrid

N-Nenzyl-piperidinyl-aryl-acylhydrazone derivatives-donepezil hybrids

NSAID-Donepezil conjugates

Phenothiazine/Donepezil-like Hybrids

Phenylpyridazine-3-carboxamide-benzylpiperidine hybrid

Phthalazin-1(2H)-one-donepezil hybrid.

Phthalimide-dithiocarbamate hybrids

Pyrazole-benzylpiperidine derivative

Pyridine-donepezil hybrids

Pyrrolizine–benzylpiperidine hybrid.

Quinolone–benzylpiperidine derivative

Racemic Trans Propargylamino-Donepezil

Tenuazonic-donepezil hybrids

Thiazole-benzylpiperidine scaffolds

Vilazodone-Donepezil chimeras

Doxycycline

(E)-5,6-dimethoxy-2-(4-(4-substituted piperazin-1-yl)benzylidene)-2,3-dihydro-1H-inden-1-ones

Ebselen-piperidine hybrids

Epigenetic drugs

Bromodomain and Extra-Terminal Domain (BET) Inhibitors

DNA Methyltransferase (DNMT) Inhibitors

Histone Acetyltransferase (HAT) Activators

Histone Deacetylase (HDAC) Inhibitors

AMX0035

Sirtuin Activators

Zinc-dependent deacetylases

Smilagenin

Ferulic acid derivatives

Ferulic acid-based 1,3,4-oxadiazole hybrids

Ferulic acid-O-alkylamine derivatives

Flavone-Cyanoacetamide hybrids

Flavonoid-N, N-dibenzyl(N-methyl)amine hybrids

6,7-Dimethoxychromone – DBMA

Fluoren-9-Amines

Galantamine derivatives

Galantamine-Curcumin hybrids

Galantamine-Memantine hybrids

Genistein-O-alkylamines derivatives

Ginkgo biloba extracts

EGb761

Glycogen synthase kinase-3 (GSK-3) inhibitors

Heptamethylene-linked levetiracetam-huprine

Heterodimeric isoindoline-1,3-dione derivatives

Histamine H3 receptor ligands

2-(5-(Azepan-1-yl)pentyloxy)-9H-xanthen-9-one

2-(5-(Azepan-1-yl)pentyloxy)-7-chloro-9H-xanthen-9-one

Biphenylalkoxyamine derivatives-Histamine H3 receptor ligands

Chlorophenoxy derivatives-Histamine H3 receptor ligands

Cyanobiphenyls

Guanidines

Non-imidazole histamine H3 receptor ligands

1-[2-thiazol-5-yl-(2-aminoethyl)]-4-n-propylpiperazine

1-[2-thiazol-4-yl-(2-aminoethyl)]-4-n-propylpiperazine

1-phenoxyalkyl-4-(amino)alkylopiperazine

Methyl(4-phenylbutyl){2-[2-(4-propylpiperazin-1-yl)-1,3-thiazol-5-yl]ethyl}amine

Huperzine A

Huprine Y–capsaicin hybrids

Hyaluronan-Carnosine conjugates

Hybrid 8-Hydroxy Quinoline-Indole derivatives

Ibudilast

Icos-huprine hybrids

Indanone derivatives

Substituted benzylidene indanone derivative

Indazole ethers

Indolylpropyl benzamidopiperazines derivates

Isolinderalactone

Kinesin MKLP-2 inhibitors

Ladostigil

Levetiracetam-huprine hybrid

Levetiracetam-(6-chloro)tacrine

Lipoic acid derivatives

Lipoic acid-N-benzylpiperidine

Lipoic acid-N,N-dibenzyl(N-methyl)amine

LM-031

Lithium

M30

Melatonin-benzyl pyridinium bromide derivates

Melatonin-Cinnamate hybrids

Memantine derivatives

Tacrine-Adamantanes Hybrids

Galantamine-Memantine hybrids

Aminoadamantane-Carbazole/Tetrahydrocarbazole hybrids

Memantine-Antioxidant hybrids

Memantine-Ferulic Acid hybrids

Memantine-Glutathione/Lipoic Acid hybrids

Memantine-Polyamine Conjugates

H2S-Releasing Memantine Prodrug

Dual P2X7-NMDA Receptor Antagonists

Memantine-Vitamin D hybrid

Memoquin

Memoquin-Lipoic acid hybrids

Meserine

(-)-Meptazinol-Melatonin hybrids

Multifunctional iminochromene-2H-carboxamide derivatives

Multifunctional thioxanthone derivatives

Multitarget-directed oxoisoaporphine derivatives

N-(2-((4,6-di(1,4,7,10-tetraazacyclododecan-1-yl)-1,3,5-triazin-2-yl)amino)ethyl)-2-oxo-2H-chromene-3-carboxamide

N-Alkylpiperidine carbamates

N-Benzylpyrrolidine derivatives

N-Cyclohexylimidazo[1,2-a]pyridine derivatives

N, N-Dibenzyl(N-methyl)amine hybrids

Neohesperidin

New phthalimide and saccharin derivatives

(26, [2-(2-(4-benzylpiperazin-1-yl)ethyl)benzo[d]isothiazol-3(2H)-one 1,1-dioxide]; 52, 2-(2-(3-(3,5-difluorobenzylamino)piperidin-1-yl)ethyl)isoindoline-1,3-dione)

Nosustrophine

N-Propargylpiperidines with naphthalene-2-carboxamide/naphthalene-2-sulfonamide moieties

Phenothiazine derivatives

Toluidine blue O (TBO) and thionine (TH)

Phenyl sulfonyl-pyrimidine carboxylate derivatives

Phosphazine and phosphazide derivatives

Phosphodiesterase 2 inhibitors

Polysubstituted Pyrazine derivatives

Compound A3B3C1

Pregnenolone derivatives

Protriptyline

Pterostilbene-O-acetamidoalkylbenzylamines derivatives

Pyrimidine/Pyrrolidine-Sertraline based hybrids

Pyrimidinylthiourea derivatives

Pyrrolo[2,3-b] quinolin-4-amine derivatives

Quinazoline derivatives

Quinazolinone derivatives

Quinazolinone-based hydrazones

Racemic benzochromenopyrimidinetriones

13-aryl-2,3,4,13-tetrahydro-1H,12H-benzo[6,7]chromeno[2,3-d]pyrido[1,2-a]pyrimidine-7,12,14-triones

15-aryl-8,9,10,11,12,15-hexahydro-14H-benzo[6',7']chromeno[2',3:4,5] pyr-imido [1,2-a]azepine-5,14,16-triones

13-phenyl-2,3,4,13-tetrahydro-1H,12H-benzo[6,7]chromeno[2,3-d]pyrido[1,2-a]pyrimidine-7,12,14-trione

Ranitidine analogs

Reserpine

Resveratrol derivatives

Deferiprone-Resveratrol hybrids

Geranylated resveratrol derivatives

Prenylated Resveratrol derivatives

Isoprenylation-Resveratrol dimer derivatives

Pyridoxine-Resveratrol hybrids Mannich base derivatives

Resveratrol-Clioquinol hybrids

Resveratrol-Maltol hybrids

Resveratrol-Tacrine hybrids

Resveratrol-Thiophene hybrids

Rhein-huprine hybrids

Rivastigmine derivatives

4'-Aminochalcone-rivastigmine hybrids

Rivastigmine-hydroxycinnamic acid hybrids

RS-0406

Salicyladimine derivatives

Scutellarein-O-acetamidoalkylbenzylamines

SKF-64346-N-Methyl-N-benzylamines hybrids

Small-molecule chaperones

Iododiflunisal

Luteolin

Sulindac

Olsalazine

Flufenamic acid

Spirocyclic alkaloids and polyphenols

Tacrine-based multi-target drugs

7-Methoxytacrine-adamantylamine heterodimers

9-Amino-1,2,3,4-tetrahydroacridine derivatives with 2-fluorobenzoic acid or 3-fluorobenzoic acid moiety

Bis-(7)-Tacrine derivatives

Capsaicin-Tacrine hybrids

Conjugates of tacrine with 1,2,4-thiadiazole derivatives

Conjugates of tacrine and salicylamide

Salicylimine derivatives

Cystamine-tacrine dimer

Cystamine-bis-(7)-tacrine hybrid

Ferulic (Lipoic) acid plus Melatonin-modified Tacrines

Methylene-linked 1,2,3,4-tetrahydrobenzo[h][1,6]naphthyridine-6-chlorotacrine hybrids

Multifunctional tacrine-donepezil hybrids

Phenylthiazole-tacrine hybrids

Quinolinetrione-tacrine hybrids

Tacrine–4-oxo-4H-chromene hybrids

Tacrine-based cyclopentapyranopyridine- and tetrahydropyranoquinoline-kojic acid derivatives

Tacrine-Adamantanes hybrids

7-MEOTA-amantadine hybrids

6-Chlorotacrine-memantine hybrid

Tacrine-Benzene/pyridine hybrid

Tacrine-Benzofuran hybrids

Tacrine–Benzotiazole derivatives

Tacrine–Bifendate hybrids

Tacrine Conjugates with 2-Arylhydrazinylidene-1,3-Diketones

Tacrine-deferiprone hybrids

Tacrine-Donepezil hybrids

Tacrine-Ferulic acid hybrids

THA–ferulic acid hybrids

THA–ferulic acid–NO-donor thihybrids

THA–ferulic acid hybrids with piperazine linker

Tacrine-flavonoid hybrids

Tacrine hybrids with HDAC Inhibitors

THA (6-Cl-THA)–HDAC inhibitors hybrids

Tacrine Hybrids with Thio Derivatives

Tacrine Hybrids with Fluorescent Probes

6-Cl-THA-5-phenylpyrano [3,2-c]quinoline hybrids

6-Cl-THA–tetrahydrobenzo[h][1,6]naphthyridine

THA–lophine hybrids

Tacrine Hybrids with Ca2+ Channel Blocker

THA–nimodipine hybrids

THA–dihydropyridine hybrid

THA-dihydropyirimidine-thione hybrids

Tacrine Hybrids with Modulators of Serotonin Receptors

THA-1-(phenylsulfonyl)-4-(piperazin-1-yl)-1H-indole hybrids

THA–5HT6-agonist hybrids

THA–Vilazodone hybrids

Tacrine Hybrids with Modulator of Muscarinic Receptors

Gallamine–THA hybrids

THA–xanomeline hybrids with amine linker

7-MEOTA–BQCA hybrids

THA–BQCA hybrids

6-Cl-THA–BQCA hybrids

THA–xanomeline hybrids

Tacrine Hybrids with Cannabinoid CB1 Receptor Antagonists

Tacrine Hybrids with Modulator of NMDA Receptors

7-MEOTA–adamantylamine hybrids

Benzohomoadamantane–6-Cl-THA hybrids

Tacrine Hybrids with Modulators of Opioid Receptors

THA–Tianeptine hybrids

Tacrine Hybrids with MAO Inhibitors

THA–selegiline hybrids

Tacrine Hybrids with Natural Products

THA–coumarin hybrids

THA–flavonoid hybrids

Huprine Y-rhein hybrids

THA-carbazoles hybrids

6-Cl-THA–Scutellarin hybrids

THA–resveratrol hybrids

THA hybrids with natural-based D-xylose, D-ribose, and and D-galactose

Tacrine-Hydroxybenzoyl-Pyridone hybrids

Tacrine-Hydroxyphenylbenzimidazole hybrids

Tacrine–Huprine hybrids

Tacrine-Ibuprofen hybrids

Tacrine-Isatin Schiff base hybrid derivatives

Tacrine–Melatonin Hybrids

Tacrine-Nimodipine hybrids

Tacrine-Resveratrol fused hybrids

Tacrine-Salicylimine derivatives

Tacrine-Scutellarin Hybrids

Tacrine-Selegiline Hybrids

Tacrine–Phenothiazine hybrids

Tacrine-1-trifluoromethoxyphenyl-3-(1-propionylpiperidin-4-yl) urea (TPPU) hybrids

6-Cl-THA–TPPU

Huprine–TPPU hybrids

Tacrine-(β-Carboline) hybrids

Tacripyrines

THA–antioxidant CR-6 hybrids

THA–(b-carbolines (pyrido [3,4-b]indoles) hybrids

THA–Caffeic acid hybrids

THA–Ebselen hybrids

THA–huperzine A hybrids

Huprine A

THA hybrids with N,N-dimethylated flavonoids

THA hybrids with NO-donating moieties

THA–(hydroxybenzoyl-pyridone) hybrids

THA–hydroxyquinoline hybrids

THA-NSAID hybrids

THA–flurbiprofen hybrids

THA–flurbiprofen–NO–donating hybrids

THA–indometacine hybrids

ROS–responsive ibuprofen–THA hybrids

THA–phenolic acid dihybrids

THA–phenolic acid–ligustrazine trihybrids

THA–Silibinin hybrids

THA–Triazole–chalkone conjugates

THA–Trolox hybrids

THA derivatives (miscelaneous)

Huprine Y derivatives

Huprine–based hybrids with 2-(2-oxopyrrolidin-1-yl)butyramide moiety of levetiracetam

THA-tryptophan hybrids

THA-indole hybrids

THA-based hybrids with anacardic acid, Cardanol and Cardols

THA-trimethoxybenzene hybrids

Photoswitchable hybrids

Quinone–THA hybrids

THA-propargylamine hybrids

7-MEOTA-p-anisidine hybrids

THA-1,2,3-triazole hybrids

Schiff base hybrids

THA-pyrimidone hybrids

THA–carbamate hybrids

THA hybrids with phosphorus moieties

Tadalafil derivates

Tenuazonic Acid derivatives

Tetrahydroisoquinoline derivatives

Dauricine

Jatrorrhizine

1MeTIQ

THICAPA

Tetrahydropyrazino-annelated theophylline (1,3-dimethylxanthine) derivatives

Thiazolidinediones

Pioglitazone

Thiosemicarbazones

Pyridoxal 4-N-(1-benzylpiperidin-4-yl)thiosemicarbazone

Toluidine blue O

Triazine-triazolopyrimidine hybrids

Tryptamine derivatives

Tryptanthrin Derivatives with Benzenesulfonamide Substituents

Vafidemstat (ORY-2001)

Xanthone derivatives

β-Carboline derivatives
