# Supplementary material for: Therapeutic Options in Alzheimer’s Disease: From Classic Acetylcholinesterase Inhibitors to Multi-Target Drugs with Pleiotropic Activity
Source: Life (Basel). 2024 Nov 26;14(12):1555. doi: 10.3390/life14121555 (PMC11678002; doi:10.3390/life14121555)
Supplement: Supplementary file 1 [file life-14-01555-s001.zip › life-3304553-supplementary/Table S5.docx]

**Table S5. Natural bioproducts with potential MT activity in Alzheimer’s disease models**

1,2,3,4,6-Penta- O-galloyleta-d-glucopyranose

10-Gingerol

1-methyl-4-(1- Methylethylideme)

2’,4’-Trihydroxychalcone

2-Hydroxysalignarine-E

3,4-Dimethoxycinnamic acid

3,4-di-o-Caffeoylquinic acid

4-Hydroxy-3-methoxyphenyl-yl-2-butanone

5-Hydroxymethyl Furfural

6-Gingerol

6-Shogaol

7-O-methyl Wogonin

Acridones from Atalantia monophyla

Citrusinine II

Ajmalicine

Allium sativum

Aminoglucosyl- glycerolipids

Angelica gigas

Angelica sinensis derivatives

Anthocyanins

Apigenin

Arecaidine

Arecoline

Aspidofractinine-3-methanol

Astaxanthin

Axillaridine-A

Ba wei di huang wan (BWDHW)

Bacopasaponin

Bacoside A

Bakuchiol derivatives

Bavachromene

Berberine

Bilobalide

Bisdemethoxycurcumin

Bogenines

Brahmine

Bulbocodin D

Buxakashmir-amine

Camellia sinensis

Camphene

Camphor

Cannabidiol

Carbazole alkaloids

Cardamonin

Carnosol

Catapols

Catechins

Catharanthine

Cerebrosides

Chaihu Shugan San

Chalcone derivatives

Chalcone-O-carbamate derivatives

2'-Hydroxychalcones

Chrysin derivatives

Cineole

Cinnamic acid

Coptis chinensis

Coumarin hybrids

Crocin

Crocus sativus

Cyclohexen

Danggui shaoyao san Huanglian jiedu tang (HLJDT)

Demethoxycurcumin

Desmodin

*Dictyostelium discoideum* derivatives

Dictyoquinone

Dictyosterol

Discodiene

Discoidol

PQA-11

PQA-18

Pt-4

Dieckol

Dihydrojatamansin)

Diplazium esculentum.

Docosahexaenoic acid

Ecliptae Herba derivatives

Ellagic acid

Epigallocatechin-3-gallate

Epimedii Herba derivatives

Eugenol

Evodiamine

Flavokawain B

Flavonoid-N, N-dibenzyl(N-methyl)amine hybrids

Gangetin

Gangetinin

Genistein-O-alkylamines derivatives

Giloin

Ginger

Ginkgetin

Ginkgo biloba extracts

Ginkgolide

Ginsenosides

Glycosaminoglycans from Litopenaeus vannamei

Glycyrrhizin

Guggulsterones

Hayatidine

Hayatine

Hengqing II decoction

Herpestine

Hesperetin derivatives

7-O-((1-(3-chlorobenzyl)-1H-1,2,3-triazol-4-yl)methyl)hesperetin

7c (7-O-(4-(Morpholinoethyl)-acetamide) hesperetin

Humulene

Hydroxycinnamic acids

Caffeic acid

Ferulic acid

Hydroxytyrosol

Isolinderalactone

Isomangiferin

Isorhapontigenin

Jatamansic acid

Jatamansine

Jatamansinol

Jatamansinone

Jatamansone

Jiawei wen dan tang

Jia-Wei-Qi-Fu-Yin

Kai-Xin-San

Kojic acid dimer

L-Arctigenin

Linalool

Linalyl acetate

Lipoic acid derivatives

Magnoflorine

Manusumbionic acid

Marine compounds

Ochrophyta:

Ecklonia cava

Dieckol

PFF-A

6,6-Bieckol

8,8’-Bieckol, Eckol

Ecklonia stolonifera

24-hydroperoxy 24-vinylcholesterol

Eckstolonol

Eckol

PFF-A

Dieckol

2-Phloroeckol

7-Phloroeckol)

Ishige okamurae

6,6’-bieckol

Eisenia bicyclis

Phlorotannin

Sargassum siliquastrum

Sargachromanol I

Sargachromanol G

Ecklonia maxima

Sulfated polysaccharides

Saccharina latissimi

Hexadecanoic acid

Fucus guiryi

Cis-9-octadecenoic acid

Sargassum sagamianum

(5E, 10Z)-6,10,14-trimethylpentadeca-5,10-dien-2,12-dione

(5E,9E, 13E)-6,10,4-trimethyl- pentadeca-5,9,13-trien-2,12-dione

Sargassum serratifolium

Sargahydroquinoic acid

Sargachromenol

Sargaquinoic acid

Padina gymnospora

Alpha-bisabolol

Hizikia fusiformis

Glycyrrhizin

18α-Glycyrrhetinic acid

18β-Glycyrrhetinic acid

Ecklonia stolonifera

Fucosterol

Phloroglucinol

Triphlorethol-A

Ishige okamurae

Diphlorethohydroxycarmalol

Phloroglucinol

Saccharina latissimi

Fucoxanthin

Rhodophyta

Gelidiella acerosa

Phytol

Gloiopeltis furcate

2-(3-Hydroxy-5-oxotetra-hydrofuran-3-yl)acetic acid

Glutaric acid

Succinic acid

Nicotinic acid

(E)-4-hydroxyhex-2-enoic acid

7-Hydroxycholesterol

Uridine

Glycerol

5-(Hydroxymethyl)-2-methoxybenzene-1,3-diol

(5E,7E)-9-oxodeca-5,7-dienoic acid

(Z)-3-ethylidene-4-methylpyrrolidine-2,5-dione Dehydrovomifoliol

Loliolide

Cholesteryl stearate

Palmitic acid

Cis-5,8,11,14,17-eicosapentaenoic acid

α-linolenic acid

Gelidium pristoides

Sulfated polysaccharides

Chlorophyta

Ulva rigida

Sulfated polysaccharides

Porifera

Latrunculia biformis

Discorhabdin G

3-Dihydro-7,8-dehydrodiscorhabdin C)

Latrunculia bocagei

Discorhabdin B Discorhabdin L

Geodia barretti

Barettin

8,9-Dihydrobarett

Bromoconicamin

Axinella verrucosa

Bromo-pyrrole alkaloid

Acanthodendrilla sp.

Homoaerothionin

Fistularin)

Acanthodendrilla sp.

Subereamolline C

Subereamolline D

Aerothionin

Cnidaria

Coral

Pseudozoanthoxanthin

Chordata

Synoicum pulmonária

Pulmonarin B

Didemnun sp.

Lepadin I

Matairesinol

Matrine

Melissa officinalis

Natural alkaloids

Indole alkaloids

Ajmalicine

Reserpine

β-Carboline alkaloids

Harmine

Harmaline

Protoberberine alkaloids

Berberine

Palmatine

Benzophenathridine alkaloids

Avicine

Nitidine

Chelerythrine)

Marine alkaloids

Imidazole alkaloids

Pseudozoanthoxanthin

Stevensine

Bromo-pyrrole alkaloids

Stevensine

Hymenialdisine

Indole alkaloids

Meridianins A-E)

Nitrogen-containing Marine Compounds

Pulmonarin

Quinazoline-Benzodiazepine alkaloids

Circumdatin D

Neohesperidin

Nicotine

Oleuropein

Pectins

Pentagalloyl glucose

Petroselinic acid

Phlorofucofuroeckol-A

Pholidota cantonensis

Phyllembelin

Phytol

Prenylated Compounds from Psoralea Fructus

Prosapogenin III

Pseudoephedrine

Pterocarpan

Pterocarpanoids

Ptychopetalum olacoides

p-Tyrosol

Pyrimidines

*Albizzia Julibrissin*

2-amino-3-ureidopropanoic acid (Albizziin)

*Tadehagi Triquetrum*:

5-(4-[(methylcarbamoyl) amino]-2-oxopyrimidin-1(2H)-yl

Tetrahydrofuran-2-Yl

Methyl Methylcarbamate

*Annona foetida*

Pyrimidine-β-carboline alkaloids

N-hydroxy annomontine

Annomontine)(anticholinesterase

*Heterostemma brownii*

6-Methoxy-4-(N-methylamino)-2-(N,N-dimethylamino)-5-(N methylformamido)pyrimidine

6-methoxy-2,4-bis(N-methylamino)-5-(N-methylformamido)pyrimidine

2-amino-6-methoxy-4-(N-methylamino)-5-(N-methylformamido)pyrimidine Heteromine A

Heteromine B

*Kirkpatrickia varialosa*

9-Amino-5-(2-aminopyrimidin-4-yl)pyrido (Variolin analogs)

*Dichroa febrifuga*

3-{3-[(2R,3S)-3-Hydroxypiperidin-2-yl]-2-oxopropyl}quinazolin-4(3H)-one (Febrifugine);

*Alchornea javanensis*

Hexahydroimidazo [1,2-a]pyrimidine (Alchorneine; Alchornidine)

*Glycyrrhiza uralennsis*

3-methy-6,7,8-trihydro-pyrrolo [1,2-a]pyrimidine-2-one (Uridine)

*Acanthostrongylophora Ingens*

Pyrimidine-β-carboline alkaloids

Acanthomine A

Ingenines A and B

*Eudistoma vannamei*

1-(4-Hydroxy-5-hydroxymethyl-tetrahydro-furan-2-yl)-5-methyl-1H-pyrimidine-2,4-dione (Staurosporine)

*Monanchora arbuscula*

3-Nonyl-6,7-dihydro-5H-pyrrolo [1,2-c]pyrimidin-1-ylideneamine

8-Butyl-7-methyl-4,5-dihydro-1H-cyclopenta[de]quinazolin-2-ylideneamine (Monalidine A; Arbusculidine A)

Resveratrol derivatives

Deferiprone-Resveratrol hybrids

Resveratrol-Maltol and Resveratrol-Thiophene hybrids

Resveratrol nanoconjugates

Resveratrol-Clioquinol hybrids.

Rhapontigenin

Rhein-Huprine hybrids

Rhinella arenarum skin derivatives

Rosavins

Rosmarinic acid

Rutin

Safranal

Safranin

Safrole

Salidroside

Salignenamide-E

Saponins

Sarsasapogenin

*Schisandra chinensis* derivatives

(E)-9-Isopropyl-6-Methyl-5,9-Decadiene-2-One

1-Phenyl-1,3-Butanedion

Nootkatone

Phenyl-2-Propanone

Scoponin

Selaginella derivatives

Serpentine

Sesamin

Sesaminol

Seselin

Sitoindoside IX

Sitoindoside X

Sitosterol

Tannins

Tanshinone

Tenuazonic acid derivatives

Tetrahydroisoquinoline derivatives

Dauricine (4-[[(1R)-6,7-dimethoxy-2-methyl-3,4-dihydro-1H-isoquinolin-1-yl]methyl]-2-[4-[[(1R)-6,7-dimethoxy-2-methyl-3,4-dihydro-1H-isoquinolin-1-yl]methyl]phenoxy]phenol)

Dauricine-Graphene oxide

**Jatrorrhizine** (2,9,10-trimethoxy-5,6-dihydroisoquinolino[2,1-b]isoquinolin-7-ium-3-ol

**1MeTIQ** (1-Methyl-1,2,3,4,-tetrahydroisoquinoline)

**THICAPA** (3-[[(3S)-1,2,3,4-tetrahydroisoquinoline-3 carbonyl]amino] propanoic acid)(N-[(3S)-1,2,3,4 tetrahydroisoquinolin-3-ylcarbonyl]-beta-alanine)

Thamnolia vermicularis

Thujone

*Tinospora sinensis* derivatives

Berberine

Aurantiamide acetate

N-P-coumaroyltyramine

Trans-syringin

3-Demethyl-Phillyrin

Tinosporide

Tinosporine

Umbelliferone

Valeranone

Valeriana amurensis

Withanolides

Withanols

Withanoside

Xixin Decoction

Yi gan san (YGS)

Zingerone

α-Asarone

α-Mangostin

α-Pinene

β-Asarone

β-Caryophyllene

β-Crinane Amaryllidaceae alkaloid Haemanthamine derivatives
