# Supplementary material for: Therapeutic Options in Alzheimer’s Disease: From Classic Acetylcholinesterase Inhibitors to Multi-Target Drugs with Pleiotropic Activity
Source: Life (Basel). 2024 Nov 26;14(12):1555. doi: 10.3390/life14121555 (PMC11678002; doi:10.3390/life14121555)
Supplement: Supplementary file 1 [file life-14-01555-s001.zip › life-3304553-supplementary/Table S6.docx]

**Table S6. Abbreviations**

***ABAT:*** 4-aminobutyrate aminotransferase

***ABCs:*** ATP-binding cassette family

***ABCA1:*** ATP-binding cassette, sub-family A (ABC1), member 1

***ABCB1:*** ATP-binding cassette, sub-family B (MDR/TAP), member 1

***ABCC1:*** ATP-binding cassette, sub-family C (CFTR/MRP), member 1

***ABCC2:*** ATP-binding cassette, sub-family C (CFTR/MRP), member 2

***ABCC3:*** ATP-binding cassette, sub-family C (CFTR/MRP), member 3

***ABCC4:*** ATP-binding cassette, sub-family C (CFTR/MRP), member 4

***ABCC8:*** ATP-binding cassette, sub-family C (CFTR/MRP), member 8

***ABCG1:*** ATP-binding cassette, sub-family G (WHITE), member 1

***ABCG2:*** ATP-binding cassette, sub-family G (WHITE), member 2 (Junior blood group)

***ABL2:*** ABL proto-oncogene 2, non-receptor tyrosine kinase

***ACACA:*** acetyl-CoA carboxylase alpha

***ACADSB:*** acyl-CoA dehydrogenase, short/branched chain

***AGPAT2:*** 1-acylglycerol-3-phosphate O-acyltransferase 2

***AKR1A1:*** aldo-keto reductase family 1, member A1 (aldehyde reductase)

***AKR1C4:*** aldo-keto reductase family 1, member C4

***AKT1***: v-akt murine thymoma viral oncogene homolog 1

***ALDH1A1:*** aldehyde dehydrogenase 1 family, member A1

***ALDH3A1:*** aldehyde dehydrogenase 3 family, member A1

***ALPs:*** alkaline phosphatases

***AMD1:*** adenosylmethionine decarboxylase 1

***APAF1:*** apoptotic peptidase activating factor 1

***APP:*** amyloid beta (A4) precursor protein

***ARG1:*** arginase 1

***ARTs:*** ADP ribosyltransferases

***ASL:*** argininosuccinate lyase

***ASS1:*** argininosuccinate synthase 1

***ATF3:*** activating transcription factor 3

***BACE1:*** beta-site APP-cleaving enzyme 1

***BAK1:*** BCL2-antagonist/killer 1

***BAX:*** BCL2-associated X protein

***BBC3:*** BCL2 binding component 3

***BCL2:*** B-cell CLL/lymphoma 2

***BCL2L1:*** BCL2-like 1

***BCL2L11***: BCL2-like 11 (apoptosis facilitator)

***BCR-ABL:*** *BCR*-*ABL* tyrosine kinase fusion

***BDNF:*** brain-derived neurotrophic factor

***BIRC3:*** baculoviral IAP repeat containing 3

***BIRC5:*** baculoviral IAP repeat containing 5

***BLK:*** BLK proto-oncogene, Src family tyrosine kinase

***BMP2:*** bone morphogenetic protein 2

***BRCA1:*** breast cancer 1, early onset

***CASP3:*** caspase 3, apoptosis-related cysteine peptidase

***CASR:*** calcium-sensing receptor

***CAT:*** catalase

***CAV1:*** caveolin 1, caveolae protein, 22kDa

***CBS:*** cystathionine-beta-synthase

***CCDN1:*** cyclin D1

***CCL8:*** C-C motif chemokine ligand 8

***CD36:*** CD36 molecule

***CDA :*** cytidine deaminase

***CDH1:*** cadherin 1, type 1

***CDK2:*** cyclin-dependent kinase 2

***CDK4:*** cyclin-dependent kinase 4

***CDK5:*** cyclin-dependent kinase 5

***CDKN1A:*** cyclin-dependent kinase inhibitor 1A (p21, Cip1)

***CDKN2A:*** cyclin-dependent kinase inhibitor 2A

***CDKN2B:*** cyclin-dependent kinase inhibitor 2B (p15, inhibits CDK4)

***CDKs***: cyclin-dependent kinases

***CDX2:*** caudal type homeobox 2

***CFLAR:*** CASP8 and FADD-like apoptosis regulator

***CFTR:*** cystic fibrosis transmembrane conductance regulator (ATP-binding cassette sub-family C, member 7)

***CHRNA1:*** cholinergic receptor, nicotinic, alpha 1 (muscle)

***CHRNA7:*** cholinergic receptor, nicotinic, alpha 7 subunit

***CLOCK:*** circadian locomotor output cycles kaput

***COL1A1:*** collagen, type I, alpha 1

***COMT:*** catechol-O-methyltransferase

***CPS1:*** carbamoyl-phosphate synthase 1, mitochondrial

***CPT1A:*** carnitine palmitoyltransferase 1A (liver)

***CREB1:*** cAMP responsive element binding protein 1

***CRP:*** C-reactive protein

***CTNNB1:*** catenin (cadherin-associated protein), beta 1, 88kDa

***CYP1A1:*** cytochrome P450, family 1, subfamily A, polypeptide 1

***CYP19A1:*** cytochrome P450, family 19, subfamily A, polypeptide 1

***CYP1A2:*** cytochrome P450, family 1, subfamily A, polypeptide 2

***CYP1B1:*** cytochrome P450, family 1, subfamily B, polypeptide 1

***CYP2A6:*** cytochrome P450, family 2, subfamily A, polypeptide 6

***CYP2C8:*** cytochrome P450, family 2, subfamily C, polypeptide 8

***CYP2C9:*** cytochrome P450, family 2, subfamily C, polypeptide 9

***CYP2C19:*** cytochrome P450, family 2, subfamily C, polypeptide 19

***CYP2D6:*** cytochrome P450, family 2, subfamily D, polypeptide 6

***CYP2E1:*** cytochrome P450, family 2, subfamily E, polypeptide 1

***CYP2J2:*** cytochrome P450 family 2 subfamily J member 2

***CYP3A4:*** cytochrome P450, family 3, subfamily A, polypeptide 4

***CYP3A5:*** cytochrome P450, family 3, subfamily A, polypeptide 5

***CYP4B1:*** cytochrome P450, family 4, subfamily B, polypeptide 1

***CYP4F2:*** cytochrome P450, family 4, subfamily F, polypeptide 2

***CYP7A1:*** cytochrome P450, family 7, subfamily A, polypeptide 1

***DAPK1:*** death-associated protein kinase 1

***DCK:*** deoxycytidine kinase

***DIO2:*** iodothyronine deiodinase 2

***DNMT1:*** DNA (cytosine-5-)-methyltransferase 1

***DNMT3A:*** DNA (cytosine-5-)-methyltransferase 3 alpha

***DNMT3B:*** DNA (cytosine-5-)-methyltransferase 3 beta

***DPYD:*** dihydropyrimidine dehydrogenase

***DRD4:*** dopamine receptor D4

***ECEs:*** endothelin converting enzymes

***EDN1:*** endothelin 1

***EGF:*** epidermal growth factor

***EGFR:*** epidermal growth factor receptor

***EP300:*** E1A binding protein p300

***ERBB2:*** erb-b2 receptor tyrosine kinase 2

***ERBB3:*** erb-b2 receptor tyrosine kinase 3

***ERK:*** elk-related tyrosine kinase

***ESR1:*** estrogen receptor 1

***ESR2:*** estrogen receptor 2 (ER beta)

***FAS:*** Fas (TNF receptor superfamily member 6)

***FLT1:*** fms-related tyrosine kinase 1

***FMR1:*** fragile X mental retardation 1

***FOS:*** FBJ osteosarcoma oncogene

***FOXO3:*** forkhead box O3

***FSHR:*** follicle stimulating hormone receptor

***GCLC:*** glutamate-cysteine ligase, catalytic subunit

***GNMT:*** glycine N-methyltransferase

***GPXs:*** phage tail proteins

***GRIN1:*** glutamate receptor, ionotropic, N-methyl D-aspartate 1

***GRIN2B:*** glutamate receptor, ionotropic, N-methyl D-aspartate 2B

***GSK3B:*** glycogen synthase kinase 3 beta

***GSS:*** glutathione synthetase

***GSTA1:*** glutathione S-transferase alpha 1

***GSTK1:*** glutathione S-transferase kappa 1

***GSTP1:*** glutathione S-transferase pi 1

***GSTT1:*** glutathione S-transferase theta 1

***HBB:*** hemoglobin, beta

***HBG1:*** hemoglobin, gamma A

***HDAC1:*** histone deacetylase 1

***HDAC11:*** histone deacetylase 11

***HDAC2:*** histone deacetylase 2

***HDAC3:*** histone deacetylase 3

***HDAC4:*** histone deacetylase 4

***HDAC6:*** histone deacetylase 6

***HDAC8:*** histone deacetylase 8

***HDAC9:*** histone deacetylase 9

***HDACs:*** histone deacetylases

***HFE:*** hemochromatosis

***HIF1A:*** hypoxia inducible factor 1, alpha subunit (basic helix-loop-helix transcription factor)

***HIST3H3:*** histone cluster 3, H3

***HIST4H4:*** histone cluster 4, H4

***HLA-A :*** major histocompatibility complex, class I, A

***HLA-B:*** major histocompatibility complex, class I, B

***HSD17B1:*** hydroxysteroid 17-beta dehydrogenase 1

***HSP90As:*** heat shock protein 90kDa alpha (cytosolic), class A

***HSPA8:*** heat shock 70kDa protein 8

***HTR3A:*** 5-hydroxytryptamine (serotonin) receptor 3A, ionotropic

***ICAM1:*** intercellular adhesion molecule 1

***IFNG:*** interferon, gamma

***IKK:*** I-kappaB kinase beta

***IL2:*** interleukin 2

***IL6:*** interleukin 6

***IL8:*** interleukin 8

***IL10:*** interleukin 10

***IL12:*** interleukin 12

***IL1A:*** interleukin 1, alpha

***IL1R:*** interleukin receptor

***IL12A:*** interleukin 12A

***IL23A:*** interleukin 23, alpha subunit p19

***IL12B:*** interleukin 12B

***IRS1:*** insulin receptor substrate 1

***JUN:*** jun proto-oncogene

***KDR:*** kinase insert domain receptor

***KLRK1:*** killer cell lectin-like receptor subfamily K, member 1

***LEP:*** leptin

***LEPR:*** leptin receptor

***MAGED1***: melanoma antigen family D1

***MAOA:*** monoamine oxidase A

***MAT1A:*** methionine adenosyltransferase I, alpha

***MGMT:*** O-6-methylguanine-DNA methyltransferase

***MLH1:*** mutL homolog 1

***MMP2:*** matrix metallopeptidase 2

***MMP9:*** matrix metallopeptidase 9

***MMPs:*** matrix metallopeptidases

***MSH2:*** mutS homolog 2

***MSR1:*** macrophage scavenger receptor 1

***MTND4:*** mitochondrially encoded NADH dehydrogenase 4

***MYC:*** v-myc avian myelocytomatosis viral oncogene homolog

***NAGS:*** N-acetylglutamate synthase

***NF2:*** neurofibromin 2 (merlin)

***NFKB1:*** nuclear factor of kappa light polypeptide gene enhancer in B-cells 1

***NFKB2:*** nuclear factor of kappa light polypeptide gene enhancer in B-cells 2 (p49/p100)

***NOS2:*** nitric oxide synthase 2, inducible

***NOS3:*** nitric oxide synthase 3 (endothelial cell)

***NQO1:*** NAD(P)H dehydrogenase, quinone 1

***NQO2:*** NAD(P)H dehydrogenase, quinone 2

***NR1I2:*** nuclear receptor subfamily 1, group I, member 2

***NR1I3:*** nuclear receptor subfamily 1, group I, member 3

***NR3C1:*** nuclear receptor subfamily 3, group C, member 1 (glucocorticoid receptor)

***NT3:*** 3'-nucleotidase

***NTRK2:*** neurotrophic tyrosine kinase, receptor, type 2

***OTC:*** ornithine carbamoyltransferase

***P2RY2:*** purinergic receptor P2Y, G-protein coupled, 2

***PARP1:*** poly(ADP-ribose) polymerase 1

***PDGFRB:*** platelet-derived growth factor receptor, beta polypeptide

***PDGFRs:*** platelet-derived growth factor receptors

***PIK3CA:*** phosphatidylinositol-4,5-bisphosphate 3-kinase catalytic subunit alpha

***PLA2R1:*** phospholipase A2 receptor 1, 180kDa

***PLCB1:*** phospholipase C, beta 1 (phosphoinositide-specific)

***PMAIP1:*** phorbol-12-myristate-13-acetate-induced protein 1

***PON1:*** paraoxonase 1

***PRDX4:*** peroxiredoxin 4

***PRKAs:*** protein kinase family, AMP-activated

***PSEN1:*** presenilin 1

***PTGES:*** prostaglandin E synthase

***PTGS1:*** prostaglandin-endoperoxide synthase 1 (prostaglandin G/H synthase and cyclooxygenase)

***PTGS2:*** prostaglandin-endoperoxide synthase 2 (prostaglandin G/H synthase and cyclooxygenase)

***RARB:*** retinoic acid receptor, beta

***RASSF1:*** Ras association (RalGDS/AF-6) domain family member 1

***RB1:*** retinoblastoma 1

***RRM1:*** ribonucleotide reductase M1

***ROS1:*** ROS proto-oncogene 1, receptor tyrosine kinase

***RRM1:*** ribonucleotide reductase M1

***RRM2:*** ribonucleotide reductase M2

***RYR1:*** ryanodine receptor 1 (skeletal)

***SCD:*** stearoyl-CoA desaturase

***SCN2A:*** sodium channel, voltage gated, type II alpha subunit

***SCNs:*** sodium channel family

***SIRT1:*** sirtuin 1

***SIRT2:*** sirtuin 2

***SIRT3:*** sirtuin 3

***SIRT5:*** sirtuin 5

***SLC5A1:*** solute carrier family 5 member 1

***SLC5A5:*** solute carrier family 5 (sodium/iodide cotransporter), member 5

***SLC6A2:*** solute carrier family 6 (neurotransmitter transporter), member 2

***SLC12A3:*** solute carrier family 12 (sodium/chloride transporter), member 3

***SLC15s:*** solute carrier family 15

***SLC19A3:*** solute carrier family 19 (thiamine transporter), member 3

***SLC22s:*** solute carrier family 22

***SLC22A16:*** solute carrier family 22 (organic cation/carnitine transporter), member 16

***SLC25A26:*** solute carrier family 25 (S-adenosylmethionine carrier), member 26

***SLC27A4:*** solute carrier family 27 member 4

***SLC28A1:*** solute carrier family 28 (concentrative nucleoside transporter), member 1

***SLC29As:*** solute carrier family 29

***SLC29A1:*** solute carrier family 29 (equilibrative nucleoside transporter), member 1

***SLCO1B1:*** solute carrier organic anion transporter family member 1B1

***SLCO1B3:*** solute carrier organic anion transporter family, member 1B3

***SMN2:*** survival of motor neuron 2, centromeric

***SNCA:*** synuclein, alpha (non A4 component of amyloid precursor

***SOCS1:*** suppressor of cytokine signaling 1

***SOCS3:*** suppressor of cytokine signaling 3

***SOD:*** superoxide dismutase

***SRC:*** SRC proto-oncogene, non-receptor tyrosine kinase

***SREBF1:*** sterol regulatory element binding transcription factor 1

***SRM:*** spermidine synthase

***STATs:*** signal transducer and activator of transcription family

***STAT1:*** signal transducer and activator of transcription 1, 91kDa

***STAT3:*** signal transducer and activator of transcription 3 (acute-phase response factor)

***SULT1C2:*** sulfotransferase family, cytosolic, 1C, member 2

***SULT1E1:*** sulfotransferase family 1E member 1

***TGFB1:*** transforming growth factor, beta 1

***TIMP3:*** TIMP metallopeptidase inhibitor 3

***TLR3:*** toll-like receptor 3

***TNF:*** tumor necrosis factor

***TNFRSF10A:*** tumor necrosis factor receptor superfamily, member 10a

***TNFRSF10B:*** tumor necrosis factor receptor superfamily, member 10b

***TNFRSF1B:*** tumor necrosis factor receptor superfamily, member 1B

***TNFSF10:*** tumor necrosis factor (ligand) superfamily, member 10

***TP53:*** tumor protein p53

***TPMT:*** thiopurine S-methyltransferase

***TRNK:*** mitochondrially encoded tRNA lysine;

***TRPs:*** transient receptor potential cation channels

***TYMS:*** thymidylate synthetase

***UCK1:*** uridine-cytidine kinase 1

***UCK2:*** uridine-cytidine kinase 2

***UGT1A1:*** UDP glucuronosyltransferase family 1 member A1

***UGT1A3:*** UDP glucuronosyltransferase family 1 member A3

***UGT1A4:*** UDP glucuronosyltransferase 1 family, polypeptide A4

***UGT1A6:*** UDP glucuronosyltransferase 1 family, polypeptide A6

***UGT1A8:*** UDP glucuronosyltransferase 1 family, polypeptide A8

***UGT1A9:*** UDP glucuronosyltransferase 1 family, polypeptide A9

***UGT1A10:*** UDP glucuronosyltransferase 1 family, polypeptide A10

***UGT2B1:*** UDP glucuronosyltransferase 1 family, polypeptide B1

***UGT2B7:*** UDP glucuronosyltransferase 2 family, polypeptide B7

***VCAM1:*** vascular cell adhesion molecule 1

***VEGFA:*** vascular endothelial growth factor A

***VEGFs:*** vascular endothelial growth factor family

***VHL:*** von Hippel-Lindau tumor suppressor, E3 ubiquitin protein ligase

***ZNF350****: zinc finger protein 350*
